# Association genetics in *Populus* reveals the interactions between Pt-miR397a and its target genes

**DOI:** 10.1038/srep11672

**Published:** 2015-06-26

**Authors:** Jinhui Chen, Beibei Chen, Xiaohui Yang, Jiaxing Tian, Qingzhang Du, Deqiang Zhang

**Affiliations:** 1 National Engineering Laboratory for Tree Breeding, College of Biological Sciences and Technology, Beijing Forestry University, No. 35, Qinghua East Road, Beijing 100083, P. R. China; 2 Key Laboratory of Genetics and Breeding in Forest Trees and Ornamental Plants, Ministry of Education, College of Biological Sciences and Technology, Beijing Forestry University, No. 35, Qinghua East Road, Beijing 100083, P. R. China

## Abstract

Recent studies have revealed associations between single nucleotide polymorphisms (SNPs) in microRNA (miRNA) genes and diseases. However, association studies to decipher the interactions between miRNAs and their target genes remain to be conducted. Here, we investigated the association of growth and wood traits with SNPs in *Pt-miR397a* and its targets, in 261 individuals from a natural population of *Populus tomentosa*. Of the 57 SNPs identified in *Pt-miR397a*, three strongly affect its secondary stability, and SNPs in target sites in *Pt-LAC20* and *Pt-HSP40* changed the binding affinity of Pt-miR397a. Single-SNP association analysis revealed that SNPs in *Pt-miR397a* significantly associated with α-cellulose content and stem volume, and SNPs in target genes also associated with growth and wood-property traits. Multi-SNP association analysis with additive and dominant models found that SNPs in six potential target genes associated with at least one trait in common with *Pt-miR397a*, revealing a possible genetic interaction between *Pt-miR397a* and its targets. Furthermore, epistasis analysis revealed epistatic interactions between SNPs in *Pt-miR397*a and its target genes. Thus, our study indicated that SNPs in *Pt-miR397a* and six target genes affect wood formation and that association studies can reveal the interactions between miRNAs and their target genes.

MicroRNAs (miRNAs) are conserved, single-stranded, noncoding RNAs that regulate the expression of target genes involved in many processes^1^,^2^. Single nucleotide polymorphisms (SNPs) commonly occur in miRNA genes, including in the regions flanking the miRNA genes (pri-miRNA), the precursor-miRNA (pre-miRNA), the mature miRNA region, and the miRNA binding sites of target genes in human^3^, animal^4^, and plant^5^. Some experimental and association studies have reported that SNPs in human miRNA genes and target genes are associated with diseases^3^,^6^. Analysis of SNP databases for humans and mice showed that mutations creating or destroying putative miRNA target sites occur frequently and might be important effectors of phenotypic variation^4^. Furthermore, a resequencing study of 16 miRNA families and their 52 binding sites in *Arabidopsis thaliana* indicated that these miRNAs and their binding sites evolve under strong selective constraints^7^. Emerging work has identified many SNPs in miRNA genes and their binding sites within target genes; also, many studies have focused on identifying the roles and functions of SNPs within miRNAs and target genes.

SNPs can affect miRNA abundance and target interactions^3^,^4^. For example, SNPs, especially those in the pre-mature sequence region, may change the stability of the pre-miRNA secondary structure. SNPs can also affect miRNA biogenesis and function by modifying miRNA-target interactions. Changes in abundance of mature miRNAs caused by SNPs in miRNA genes can affect miRNA-mediated translational suppression and phenotypic variation^8^. SNPs in mature miRNAs or in their binding sites in target genes will also change the binding affinities of the miRNAs to the target mRNA, by creating or disrupting miRNA-target interactions^8^. A systematic characterization of miRNA-related SNPs indicated that SNPs in miRNA seed regions would cause the loss or gain of nearly half of the predicted targets, on average^8^. Polymorphisms in the miRNA or in the miRNA-binding sites of target genes represent genetic variation that can modulate the regulatory interaction between miRNAs and their target genes and probably affect phenotypic variation^9^. In addition to the seed region, other residues in the mature miRNA sequence or the sequences around target sites might also affect target recognition^1^. Although growing evidence shows that, as in protein-coding genes, SNPs occur in many miRNAs and may contribute to phenotypic diversity^6^, few SNPs in miRNA genes and miRNA-binding sites have been identified in plants, particularly in trees.

SNPs in miRNAs and their targets could provide a key resource for association genetics studies to explore the roles and interactions of miRNAs and their targets. Recently, many studies have used high-throughput sequencing to identify and profile stress-responsive or tissue-specific miRNAs in *Populus*^10^,^11^, a genus that includes several model and commercially important tree species^12^. Furthermore, many differentially and highly expressed miRNAs, which may play roles in the regulation of wood development in tree species, were identified from the xylem of *Populus trichocarpa*; these include Pt-miR397a^10^,^11^. However, transgenic analysis in trees remains difficult, especially for miRNAs; thus, exploring the roles of miRNAs in trees will require another strategy. Candidate gene-based SNP association has been used to identify the roles of genes associated with growth and wood properties in several tree species, including conifers^13^ and *Populus*^14^. Since SNP-based association mapping provides another approach for annotation of gene function and identification of genetic regulatory networks, it may be useful for exploration of the roles of miRNAs. Moreover, epistasis, where the function of one gene depends on the presence of a second, can be used to define functional relationships between genes and pathways, and to identify genetic regulatory networks between miRNAs and their targets^15^.

Recent work identified *P. trichocarpa* miR397a as a negative regulator of *laccase* (*LAC*) genes^16^, which are involved in lignin deposition in xylem. Suppression of *Arabidopsis AtLAC4* and *AtLAC17* resulted in reduced lignin content^17^ and transgenic studies also showed that *LAC* genes affect lignin content in *P. trichocarpa*^16^. However, which *LAC*s are regulated by Ptr-miR397a remains unknown, since the *P. trichocarpa* genome has 49 *PtrLAC*s and 30 are expressed in differentiating stem xylem, suggesting that *LAC*s function redundantly in lignin biosynthesis in wood formation^16^. In addition to *LAC*s, *HSP40* (heat shock protein 40), *LEA* (late embryogenesis abundant), and *SPRY* (SPRY receptor) are also potential targets of miR397a. However, little is known about the roles of these genes in wood formation, except that genetic and proteomics studies have found that some *HSPs* are up-regulated in the xylem of *Eucalyptus* and *Populus* species^18^. Also, *LEA*s are preferentially expressed in the late mature wood stage^19^ and a SPRY-domain gene from *Pinus radiata* may function in vascular bundle development^20^, indicating that they may play roles in cell wall formation and xylem development. Additionally, Pt-miR397a showed high abundance and differential expression in our tension wood study. Thus, miR397a and its potential targets have functions that are closely related to wood formation. To explore the functions of Pt-miR397a and its genetic regulatory interactions with target genes involved in wood formation, here we investigated the nature of genetic variation (additive, dominant, and epistatic effects) for Pt-miR397a and its six potential target genes, with nine quantitative traits, using single and multi-SNP association approaches, in *Populus tomentosa*. We identified SNPs that strongly affect the secondary stability of *Pt-miR397a* transcripts and change the binding affinity of Pt-miR397a to its targets; these SNPs also associated with growth and wood-property traits. This analysis thus provides a new strategy to examine the genetic architecture of traits involving miRNA-target interactions.

## Results

### Isolation of the *Pt-miR397a* locus and six potential targets of Pt-miR397a

To identify SNPs within *Pt-miR397a*, including the flanking regions, pre-miRNA, and mature miRNA regions, we cloned the full-length *Pt-miR397a* gene based on the sequence of ptc-MIR397a in miRbase (21). The primary *Pt-miR397a* transcript is 1,387 bp in length, and contains the 120 bp sequence of the pre-mature miRNA with the 21 bp mature sequence, and flanking sequences of 1,267 bp (KP403489-KP403528). Prediction of secondary structure by RNAfold analysis of the Pt-miR397a pre-mature miRNA sequence revealed a typical hairpin structure, confirming that Pt-miR397a is a miRNA (Fig. S1).

**Figure 1:**
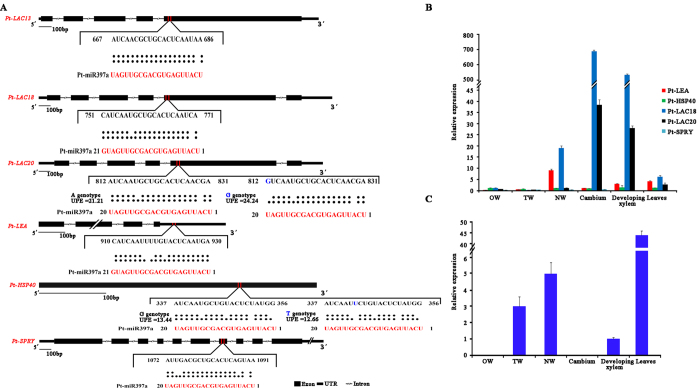
Target sites in six genes and expression of five target genes and Pt-miR397a in tissues of *P. tomentosa*. **A**: The target sites were determined by psRNATarget and SNP in *Pt-LAC20* and *Pt-HSP40* were displayed with UPE (ΔΔG, kcal/mol). **B**: The expression of five target genes in mature xylem of OW (opposite wood), TW (tension wood), NW (normal wood), cambium, developing xylem, and leaves. **C**: The expression of mature Pt-miR397a in OW, TW, NW, cambium, developing xylem, and leaves. Error bars indicate standard deviations.

We further identified 41 candidate target genes of Pt-miR397a using psRNATarget prediction (Table S1), and we used sequence-specific PCR amplification to isolate genomic DNA clones of the target sites for six genes, *Pt-LAC13* (KP403329-KP403368), *Pt-LAC18* (KP403329-KP403368), *Pt-LAC20* (KP403369-KP403408), *Pt-HSP40* (KP403289-KP403328), *Pt-LEA* (KP403449-KP403488), and *Pt-SPRY* (KP403529-KP403568) (Table 1 and Fig. 1A). Reverse transcription-quantitative PCR (RT-qPCR) analysis in xylem, cambium, and leaf samples indicated that *Pt-miR397a* expression levels and transcript levels of five of the potential target genes showed a significant, negative correlation (r(*Pt-LEA*) = −0.248, r(*Pt-HSP40*) = −0.212, r(*Pt-LAC18*) = −0.37, r(*Pt-LAC20*) = −0.328 and r(*Pt-SPRY*) = −0.467, P < 0.05; Fig. 1B,C).

### SNPs that affect the predicted secondary structure of the pre-mature sequence of Pt-miR397a and its binding affinity to its target genes

**Figure 2:**
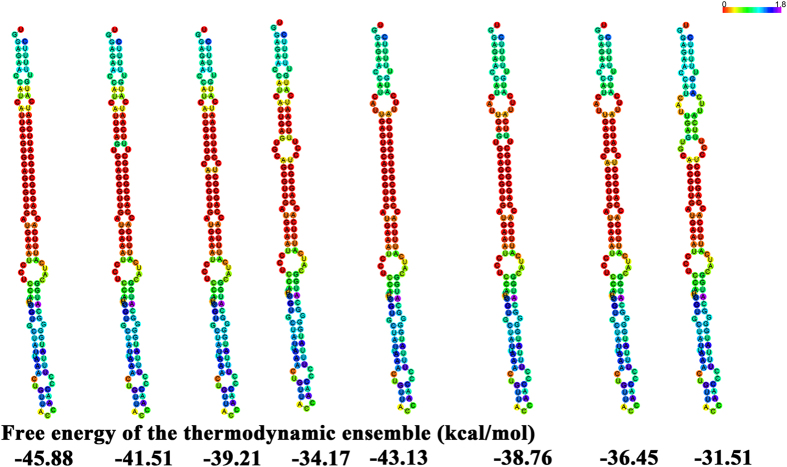
The SNPs in secondary structures of the *Pt-miR397a* pre-mature sequence with free energy of the thermodynamic ensemble (kcal/mol).

To identify SNPs, we sequenced *Pt-miR397a* and six of its targets in 40 individuals from a natural population of *P*. *tomentosa*; this detected 57 SNPs in *Pt-miR397a* (Table 1). Analysis of SNP distribution indicated that the mature region of *Pt-miR397a* was the most conserved, as we found no SNPs in it (Table 1). We found three SNPs in 99 nt of the *Pt-miR397a* pre-mature region excluding the mature region; RNAfold analysis of the miRNAs with these SNPs predicted eight miRNA secondary structures that formed different numbers of loops and had free energies of −45.88 to −31.51 kcal/mol for the pre-mature sequence of Pt-miR397a (Fig. 2). The changes in secondary structures indicate that SNPs in the pre-miRNA could strongly affect its secondary stability, potentially interfering with precursor processing and thereby greatly affecting accumulation of Pt-miR397a. In the flanking regions of *Pt-miR397a*, we also detected informative SNPs at a frequency of 1 SNP per 25 bp on average, which is more frequent than in other regions of *Pt-miR397a* (Table 1).

In the six potential target genes of Pt-miR397a, we detected 107 SNPs. Of these, we found only two SNPs in the predicted miRNA target sites (total 120 nt), *Pt-LAC20*-SNP05 (G > A) and *Pt-HSP40*-SNP05 (G > T), indicating that the target sites show high conservation. The ΔΔG (the energy required to open the secondary structure around the target site, a value that thus reflects the binding affinity) between Pt-miR397a and *Pt-LAC20* was lower for the A allele (21.21 kcal/mol) than the G allele (24.24 kcal/mol). Also, the SNP in the target site of *Pt-HSP40* changed the ΔΔG between Pt-miR397a and *Pt-HSP40*, resulting in higher energy for the G allele (13.44 kcal/mol) than the T allele (12.66 kcal/mol) (Fig. 1A). Lower energy needed to open the secondary structure around the target site means a higher possibility that the miRNA can contact (and cleave) the target mRNA. Therefore, the ΔΔG between Pt-miR397a and *Pt-LAC20* or *Pt-HSP40* suggested that Pt-miR397a has a higher binding affinity for the A genotype of *Pt-LAC20* and the T genotype of *Pt-HSP40*.

### Association analysis of allelic variation in Pt-miR397a and its targets with growth and wood properties

To explore the effects of the SNPs in *Pt-miR397a* and its targets on tree phenotypes, we conducted association tests between 164 SNPs from *Pt-miR397a* and its targets and nine growth and wood property traits, using the generalized linear model in TASSEL 2.1. Taking into account population structure, we identified 42 significant associations representing 23 SNPs in *Pt-miR397a* and its target genes (*P* < 0.01); each association explained from 7.80% (*Pt-LAC13*-SNP13) to 19.45% (*Pt-LAC13*-SNP18) (Table 2) of the phenotypic variation. Seven unique SNPs from *Pt-miR397a* (SNP18, SNP32, SNP33, SNP35, SNP38, SNP46, and SNP50) significantly associated with three phenotypic traits, α-cellulose content, tree diameter at breast height (DBH), and stem volume (V), suggesting that *Pt-miR397a* has important roles in tree growth and wood properties (Table 2).

For SNPs in the target genes, we identified thirty significant associations with nine traits. For chemical composition, one (*Pt-SPRY*-SNP08), two (*Pt-LEA*-SNP33 and *Pt-SPRY*-SNP08), and three (*Pt-HSP40*-SNP01, *Pt-LEA*-SNP29 and *Pt-LEA*-SNP32) SNPs were closely linked to holocellulose content, α-cellulose content, and lignin content, respectively (Table 2). For physical properties, *Pt-LEA*-SNP32 was significantly associated with fiber length and fiber width, *Pt-LEA*-SNP33 was significantly associated with fiber length, and *Pt-LAC20*-SNP15 and *Pt-LEA*-SNP02 were significantly associated with microfiber angle (Table 2). Most of the significant markers were associated with growth traits; for example, five SNPs in *Pt-LAC13* (SNP01, SNP12, SNP13, SNP17, and SNP18) simultaneously associated with V and DBH. Interestingly, four SNPs in *Pt-LEA* associated with microfiber angle, fiber width, H, lignin, and α-cellulose content (Table 2), providing new insight into *Pt-LEA*’s potential functions in *Populus* growth and wood formation. Among the SNPs in target genes, as expected, the SNPs in target sites were significantly associated with phenotypic traits, including *Pt-HSP40-*SNP05 (G > T) associated with tree height (H) (*P* = 0.017%) and DBH (*P* = 0.52%). Notably, *Pt-LAC13*-SNP18 (A > T, *P* = 0.012%) was significantly associated with H and explained 19.45% of the phenotypic variation, the highest contribution to phenotype.

We next used the Bayesian hierarchical model (fGWAS), emphasizing multi-SNP additive and dominant effects for each quantitative trait, to perform multi-SNP association with nine growth and wood quality traits. Under the additive and dominant effects models, we detected 89 significant associations for 54 SNPs in seven genes associated with all nine traits and found that each SNP explained 0.52 to 22.55% of the phenotypic variation (average R^2^ = 6.72%; Table S2). The total numbers of identified SNP-trait associations varied across trait categories, with 26 associations for wood chemical composition, 35 for wood physical properties, and 28 for growth traits. Twenty-four of the 54 SNP markers exhibited significant associations with at least two traits and the number of significant SNPs ranged from 5 to 18 for each trait (Table S2). Correspondingly, each trait was associated with variation in at least four candidate genes (Table S2). For wood physical properties, SNP-trait associations showed that three genes (*Pt-Hsp40*, *Pt-LEA*, and *Pt-miR397a*) associated with three traits (Table S2). Although six genes were associated with chemical composition, only *Pt-LEA* and *Pt-miR397a* each associated with three traits (Table S2). Of these, SNPs in *Pt-miR397a* and *Pt-LAC20* were associated with lignin content and *Pt-LAC18*, *Pt-LAC20*, and *Pt-miR397a* were associated with α-cellulose content (Table S2). For growth traits, additive models found all seven genes had SNPs associated with variation in growth traits; of these, *Pt-LEA* SNPs associated with the three growth traits, and with the three wood physical properties and chemical composition (Table S2). Furthermore, we found that *Pt-miR397a* was associated with eight phenotype traits, including all of the traits except H (Table S2).

**Figure 3:**
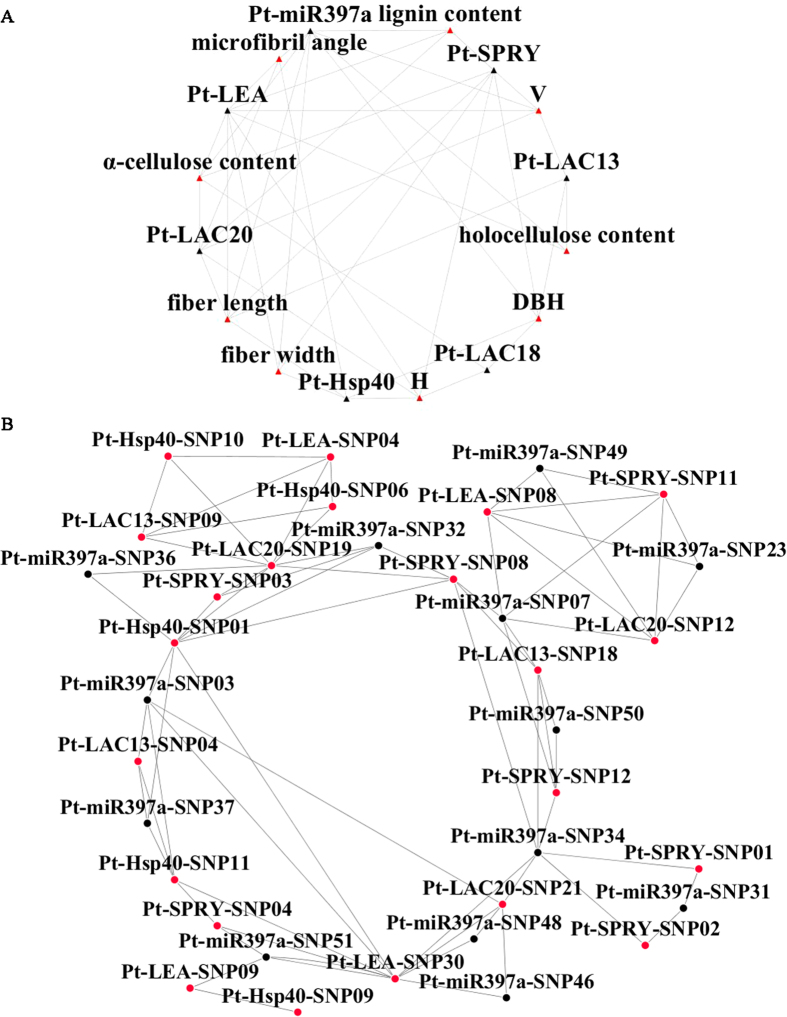
Summary of interactions between Pt-miR397a and its target genes. **A**: Association between SNPs in *Pt-miR397a* and its target genes (*Pt-LAC13*, *Pt-LAC18*, *Pt-LAC20*, *Pt-LEA*, *Pt-HSP40*, and *Pt-SPRY*) under additive and dominant models. **B**: Epistatic interactions between SNPs in *Pt-miR397a* and its target genes. Black and red triangles represent genes and traits, respectively. Black and red dots represent SNPs in *Pt-miR397a* and its target genes, respectively. Grey lines represent interactions.

Under the dominant effect model, we detected 52 associations with positive dominance values and 37 with negative values, across all three trait categories (Table S2). Many genes associated with multiple traits within/across trait categories, and we identified different SNPs with different effects from the same gene. The number of SNP associations with positive versus negative effects across the three trait categories was 17 and 9 for wood chemical compositions, 17 and 18 for wood physical properties, and 18 and 10 for growth traits (Table S2). We found eleven SNPs from four genes (*Pt-LAC18*, *Pt-LEA*, *Pt-miR397a*, and *Pt-SPRY*) that simultaneously had positive and negative dominant effects on different traits (Table S2). For example, *Pt-miR397a*-SNP48 had a negative dominant effect on fiber length, but a positive effect on tree DBH and stem V. Table S2 shows detailed descriptions of the genetic parameters for all traits with positive and negative dominant effects. In summary, SNPs in *Pt-miR397a* and its target genes (*Pt-LAC13*, *Pt-LAC18*, *Pt-LAC20*, *Pt-LEA*, *Pt-HSP40*, and *Pt-SPRY*) associated with at least one trait in common, reflecting the possible genetic interaction of the miRNA and its target genes and indicating that they may function in the same pathway (Fig. 3A).

**Figure 4:**
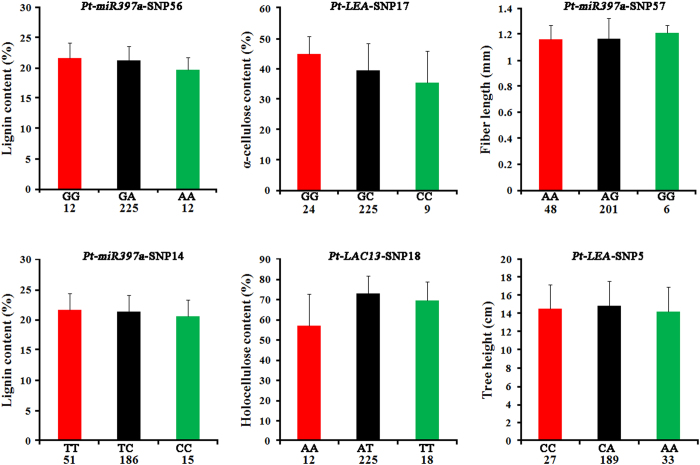
Partial genotypic effects of SNPs significantly associated with growth and wood properties. Trees were segregated into genotype categories, and corresponding phenotypes were plotted. Error bars indicate standard deviations; the x axes represent the genotype categories, and the y axes represent the traits that the SNPs are associated with: tree height, holocellulose content, α-cellulose content, lignin content, and fiber length.

To quantify the modes of gene action, we calculated the ratio of dominant (d) to additive (a) effects (|d/a|). Twenty marker-trait associations were consistent with additive effects (|d/a| ≤ 0.5), and the remaining associations were split between over- and under-dominance (|d/a| > 1.25, n = 44) and partially to fully dominant effects (0.50 < |d/a| < 1.25, n = 25). For example, the three genotypes of *Pt-LEA*-SNP17 showed significant differences in α-cellulose content (45.06% for GG, 39.79% for GC, and 35.75% for CC), consistent with additive effects (Fig. 4). Moreover, the three genotypes of *Pt-LAC13*-SNP18 showed significant differences in holocellulose content (57.30% for AA, 73.28% for AT, and 69.75% for TT), consistent with over- or under-dominance effects (Fig. 4). The average values of lignin content across genotypic classes for *Pt-miR397a*-SNP14 (21.74% for TT, 21.42% for TC, and 20.64% for CC) were suggestive of partial dominance, with the T allele being partially dominant (Fig. 4).

### Epistatic interactions between *Pt-miR397a* and its target genes

Potential epistatic interactions of the SNPs in *Pt-miR397a* and its target genes for growth and wood quality traits were examined by Multifactor Dimensionality Reduction (MDR)^21^, which can identify SNP-SNP interactions in a population. After statistical significance analysis, we identified 39 associations (*Q* ≤ 0.10) with nine traits, including 32 unique SNPs from *Pt-miR397a* and its potential target genes, (except *Pt-LAC18*) with the main effects ranging from 0.08% to 9.67% (Table S3). Analyzing the pairwise effects identified 85 SNP pairs with epistatic interactions ranging from 0.15% to 14.08% (Table S4), which were higher than the single-SNP associations (Table S3). Of the total SNP-SNP interactions, 51.6% represented miRNA-mRNA interactions, and 14.1% and 34.3% represented miRNA-miRNA or mRNA-mRNA interactions, respectively (Table S4). Multi-way SNP-SNP interaction analysis showed that the epistatic interactions among SNPs from *Pt-miR397a* and its target genes ranged from −8.73% to 6.95% (Table S4). We also detected epistatic effects among different SNPs in the same gene. For instance, we identified 12 epistasis pairs consisting of nine SNPs in *Pt-miR397a* for α-cellulose, fiber length, DBH, and H, all with negative interaction values (Table S4). Interestingly, we found two pairs that associated with two different traits. For example, *Pt-miR397a*-SNP03 and *Pt-LEA*-SNP30 associated with holocellulose and α-cellulose, and showed positive and negative interactions, respectively. Also, *Pt-miR397a*-SNP51 and *Pt-LEA*-SNP30 associated with fiber width and microfibril angle, and showed positive interactions.

**Figure 5:**
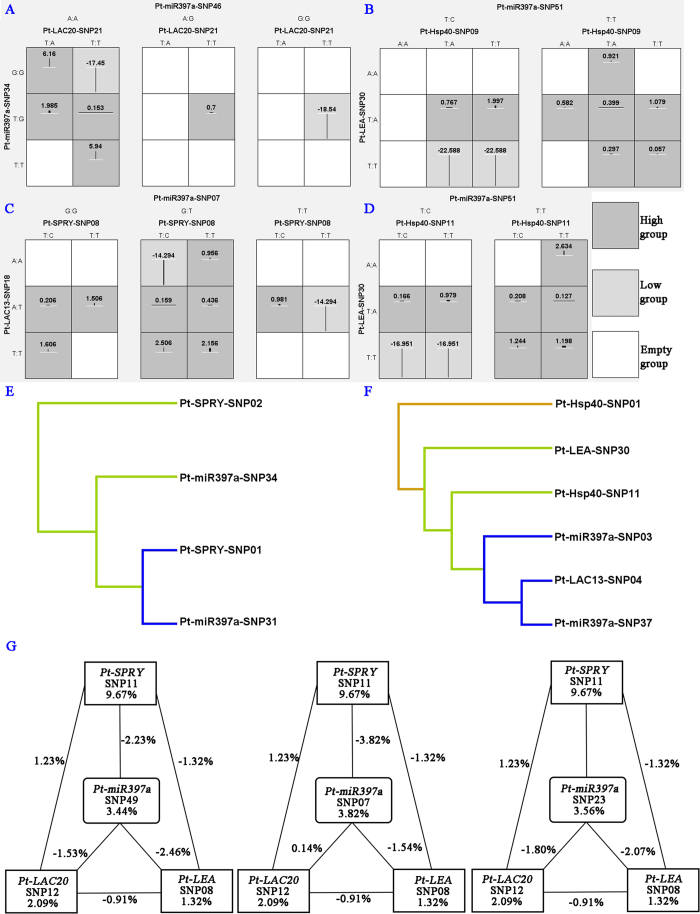
The epistatic interactions between SNPs in *Pt-miR397a* and its target genes. **A**-**D**: Distribution of empty group (white shading), high group (dark shading), and low group (light shading) combinations of *Pt-miR397a*, *Pt-SPRY*, *Pt-LAC20*, and *Pt-HSP40* genotypes associated with four phenotypes (**A**: α-cellulose content; **B**: fiber width; **C**: tree diameter at breast height; **D**: microfibril angle). **E-F**: Dendrograms of interaction among phenotype (**E**: fiber length, **F**: holocellulose content), *Pt-miR397a*, *Pt-SPRY*, *PtLAC13*, *Pt-HSP40*, and *Pt-LEA*. Blue and green represent a joint effect and blue is greater than green. Red represents an antagonistic effect. **G**: Entropy-based interaction graph for tree height among SNPs in *Pt-miR397a*, *Pt-SPRY*, and *Pt-LEA*. Values in the boxes represent the individual information gain and the positive values along the line representing positive interaction. The negative values can be explained as negative interaction/redundancy, i.e. the amount of information shared by the attributes.

The genotypic combinations associated with high and low values for α-cellulose (Fig. 5A), fiber width (Fig. 5B), DBH (Fig. 5C), and microfibril angle (Fig. 5D) are shown. The patterns of high- and low-value groups clearly differ across each of the different multilocus dimensions that were considered as evidence of epistasis, or gene-gene interactions among *Pt-miR397a*, *Pt-SPRY*, *Pt-LAC20*, and *Pt-HSP40* (Fig. 5A–D). Also, the dendrograms of interactions among phenotype, *Pt-miR397a*, *Pt-SPRY*, *Pt-LAC13*, *Pt-HSP40*, and *Pt-LEA* showed a strong joint effect between *Pt-miR397a*-SNP31 and *Pt-SPRY*-SNP01, as well as *Pt-miR397a*-SNP31 and *Pt-LAC13*-SNP04 (Fig. 5E,F). Interestingly, we detected a strong antagonistic effect between *Pt-HSP40*-SNP01 and *Pt-LEA*-SNP30 (Fig. 5F). To visualize the two-way interaction for growth and wood quality traits, we created an entropy-based interaction graph for H; this also pointed toward interactions between SNPs in *Pt-miR397a*, *Pt-LAC20*, *Pt-SPRY*, and *Pt-LEA* (Fig. 5G). All six variants explained phenotypic variation from 1.32% to 9.67% by themselves. Among the twelve interactions, we only found three positive values. Most of these were negative epistatic effects that showed redundancy between loci, indicating that these loci provide, in part, the same information for the traits^21^. For example, the two SNPs produced an additional −3.82% phenotypic variation explanation between *Pt-miR397a*-SNP07 and *Pt-SPRY*-SNP11 (Fig. 5G). We also identified negative epistatic effects between *Pt-LAC13* and *Pt-LAC20* in agreement with the postulated functional redundancy of the *LAC*s (Table S4). Notably, eleven two-locus-genotype combinations were associated with lignin content, including *Pt-miR397a*-SNP32 or *Pt-miR397a*-SNP36 with *Pt-LAC20*-SNP19 (Table S4). Taking together all the miRNA-mRNA and mRNA-mRNA (target genes) associations with the nine growth and wood quality traits, as shown in Fig. 3B, these data support the existence of epistatic interactions between *Pt-miR397a* and five of its target genes (*Pt-LAC13, Pt-LAC20*, *Pt-LEA*, *Pt-HSP40*, and *Pt-SPRY*), indicating that these genes may contribute to the same pathways in wood formation or tree growth. Considering the negative relationship between expression of Pt-miR397a and its target genes (*Pt-LAC20*, *Pt-LEA*, *Pt-HSP40*, and *Pt-SPRY*, Fig. 1B,C) that we identified by RT-qPCR and the epistatic interactions between genes, our data also indicate that negative interactions exist among them.

## Discussion

SNPs in pre-miRNA regions could change the RNA secondary structure and thus impair processing or maturation of the miRNA, eventually affecting the expression of a multitude of target genes^8^. For example, recent work showed that a common polymorphism in pre-miR-146a affects miRNA expression and contributes to a genetic predisposition to papillary thyroid carcinoma^22^. In rice, one SNP altered the abundance of the long non-coding RNA *LDMAR* (long-day-specific male-fertility-associated RNA)^23^, leading to premature programmed cell death in developing anthers. Thus, SNPs within miRNA genes can greatly change the production of the mature miRNAs, and in turn dramatically influence phenotypic variation. Similarly, the SNPs we identified in *Pt-miR397a*, particularly in the pre-miRNA region, could strongly affect its secondary stability and alter the abundance of mature Pt-miR397a. In this study, using the natural population, we found three SNPs in the pre-miRNA domains of Pt-miR397a, resulting in eight secondary structures with obvious changes in free energy of the thermodynamic ensemble (Fig. 2). However, to what extent these SNPs affect the abundance of Pt-miR397a remains unknown and will be interesting to explore in the future. Consistent with alterations in gene regulation, SNPs in *Pt-miR397a* were associated with phenotypic variation in our study, either by single SNP or multi-SNP association analysis. Thus, these SNPs in *Pt-miR397a* have potential for future research. Furthermore, we found no SNP in the mature sequence and more SNPs in the flanking regions than in the pre-mature sequence. These findings support the idea that the mature sequence of Pt-miR397a showed the most conservation, as expected, and emphasized the importance of Pt-miR397a for crucial cellular processes including the regulation of gene expression.

Target site accessibility is an important determinant of the efficacy of RNA-RNA interactions, including antisense nucleic acids, ribozymes, and miRNAs^24^ and governs miRNA-mediated regulation^14^. To measure the structural accessibility of the target site, Kertesz *et al.*^25^ performed free energy predictions, taking into account the free energy lost by mismatching target site nucleotides (ΔΔG) to estimate the binding affinity. On the basis of this model, they experimentally showed that target site accessibility is as important as sequence match in the seed region and, therefore, miRNA target sites preferentially occur in mRNA regions with high accessibility^25^. In our study, Pt-miR397a had a higher predicted binding affinity for the A and T genotypes of *Pt-LAC20* and *Pt-HSP40*, respectively, than for the G genotype, according to the ΔΔG calculated by psRNATarget. This indicates that phenotypic variation may be caused by the different affinities of Pt-miR397a for binding to *Pt-LAC20* or *Pt-HSP40* caused by SNPs, which also associated with phenotypic variation in our study (Table 2). We identified more SNPs around the target sites than in the target sites, showing that SNPs occur less frequently in the target sites than in other portions of the genome. Although the interaction between miRNA and target genes occurs via the target sites, particularly the seed region, the regions around the target sites may affect the efficiency of binding of miRNAs to target transcripts^24^. Thus, SNPs identified in the regions around the target sites may affect the recognition and binding of Pt-miR397a and its target genes. Our association study with nine growth and wood quality traits supported this supposition (Table 2).

The present study identified 42 significant associations representing 23 SNPs in the association population and these explained 7.80% to 19.45% of the phenotypic variation in the tested traits (*P* < 0.01) (Table 2). Interestingly, seven unique SNPs from *Pt-miR397a* were significantly associated with α-cellulose content, DBH, and V; this supported the idea that Pt-miR397a may have roles in wood formation and growth. These findings were in agreement with the expression pattern of *Pt-miR397a*, as identified in previous studies^10, 11^ and this study. For target gene associations, all SNPs were associated with growth traits (Table 2). *LAC*s, as important genes in lignin biosynthesis, could affect lignin content in poplar^16^, but different *LAC*s have different roles^17^. Our study suggested that *LAC*s have roles in tree growth and improved our understanding of *LAC* functions. In addition, previous studies showed that *Pt-HSP40*, *Pt-LEA*, and *Pt-SPRY* may play roles in cell wall formation and xylem development, thus potentially affecting wood formation. Here, we found that SNPs in *Pt-HSP40* and *Pt-LEA* were significantly associated with growth and wood property traits, indicating their roles in wood formation. Our association study also showed that SNP05 (G > T) in the target site of *Pt-HSP40* was associated with H and DBH (Table 2). MiRNA-binding site polymorphisms can affect human diseases by interfering with miRNA-mediated gene regulation^26^. Similarly, our study showed that a SNP within an miRNA binding site could lead to phenotypic changes, probably by altering expression of target genes.

Besides single-SNP associations, we further used fGWAS to identify pairs of SNPs associated with one trait^27^, to explore the genetic interaction between Pt-miR397a and its target genes. The sign of the allele interaction, negative or positive, provides a clue to the underlying gene regulatory mechanisms and dominant or additive gene actions may contribute to allele interaction^28^. In our study, 89 significant associations, representing 54 unique SNPs within seven genes, were associated with the nine traits (Table S2 and Fig. 3A). For lignin content, six SNPs in *Pt-miR397a* and *Pt-LAC20* had significant associations, indicating that *Pt-miR397a* and *Pt-LAC20* may contribute to the same pathway affecting lignin content, consistent with a previous study showing that Ptr-miR397a negatively regulates *LAC* genes, thus affecting lignin content in *P. trichocarpa*^16^. In addition, SNPs in *Pt-LAC13*, *Pt-LAC18, Pt-LAC20*, and *Pt-miR397a* associated with the same trait, reflecting the possible genetic interaction of *Pt-miR397a* and its target genes. Alleles with a dominant or additive effect also provide important resources for plant breeding and may prove useful for poplar breeding, especially in improvement of wood traits (Fig. 4). Our analysis clearly demonstrates that SNPs in *Pt-miR397a* and its target genes interact and may affect phenotypes via the same pathway.

In epistasis, an allele of one gene masks the phenotype of an allele of another gene. In metabolic pathways, a mutation disrupting an enzyme that acts early in a biosynthetic pathway is generally epistatic to a mutation that blocks downstream steps in the same pathway^29^. This principle has been used extensively to determine functional relationships between genes in biosynthetic and developmental pathways, as well as the contribution of alleles to quantitative traits^30^. Therefore, epistasis analysis has considerable potential to reveal interactions between genes and metabolic pathways as well as regulatory pathways and networks. Bioinformatic prediction and expression analysis showed that there are probably negative regulatory relationships between Pt-miR397a and its target genes. Consistent with this, a previous study showed the regulatory between Ptr-miR397a and *PtrLAC*s^16^. Considering that Pt-miR397a and its potential target genes are closely related to wood formation, as shown by our single-SNP and multi-SNP association in agreement with previous studies^16^,^17^,^18^,^19^,^20^, and there are regulatory links between Pt-miR397a and its target genes, epistasis analysis provides a suitable tool to explore the interactions between Pt-miR397a and its target genes.

Thus, we used MDR for identification of associations among genes whose effects occur primarily through interaction^21^, to identify epistatic interactions of *Pt-miR397a* and its target genes and their effects on wood chemical composition, physical properties, and growth traits. We identified 85 SNP pairs with epistatic interactions ranging from 0.15% to 14.08%, representing 32 unique SNPs from *Pt-miR397a* and its target genes. Also, most of the miRNA-mRNA interaction values were negative, suggesting that *Pt-miR397a* and its target genes have redundant functions^31^ and may contribute to the same pathways in wood formation and tree growth. Genes interacting via negative epistatic interactions often carry out related roles where the absence of one can be compensated for by the other, and here we found that *Pt-miR397a* and its target genes may have similar roles and function in the same pathway. Of these, importantly, we found negative interaction values in the interaction of *Pt-miR397a*-SNP32 or *Pt-miR397a*-SNP36 with *Pt-LAC20*-SNP19, which associated with lignin content (Table S4). As for the four pairs of SNPs in *Pt-miR397a* and *Pt-LAC20* associated with α-cellulose, the two-locus best gene-gene interaction model showed an interaction between *Pt-miR397a*-SNP34 or *Pt-miR397a*-SNP46 and *Pt-LAC20*-SNP21 (Fig. 5A). Further, we identified a strong interaction between *Pt-miR397a* and *Pt-LAC13* (Fig. 5F) affecting holocellulose content. Interestingly, we found no association with phenotype between *Pt-miR397a* and *Pt-LAC18*, indicating no epistatic interaction. As shown by Lu *et al.*^16^, Ptr-miR397a negatively regulates *LAC* genes; however the *LAC*s that are regulated by Ptr-miR397a were not identified. In our study, the detection of epistasis supported the interaction between *Pt-miR397a* and *Pt-LAC13* or *Pt-LAC20*, especially associated with α-cellulose, lignin content, and holocellulose content (Fig. 4B). In addition, a previous study showed functional redundancy exists in the *LAC*s^16^ and we detected negative epistatic effects that indicate redundancy between *Pt-LAC13* and *Pt-LAC20*. We found no epistatic interaction between *Pt-miR397a* and *Pt-LAC18* and whether *Pt-LAC18* could be regulated by Pt-miR397a will require additional research.

In the present study, we found significant epistatic interactions between *Pt-miR397a* and its target genes and identified negative epistatic interactions between them. Genes with similar molecular functions normally have similar profiles of epistatic interactions, suggesting that a seed set of epistatic interactions can be used to accurately predict more interactions for a given gene^32^. Also, there is extensive evidence for epistatic interactions among quantitative trait loci affecting mean genotypic values in *Drosophila* and *Arabidopsis*^33^,^34^. Knowledge of interactions between loci can be used to infer genetic networks affecting complex traits^35^, greatly informing the underlying biology. Consistent with this, a previous study revealed that the molecular mechanism underlying epistasis of tomato color mutations involves regulation of *phytoene synthase 1* expression by *cis*-carotenoid^36^. Taking into consideration the negative expression of *Pt-miR397a* and its target genes, the epistatic interactions between *Pt-miR397a* and its target genes could reflect a negative interaction. Pt-miR397a has high expression in xylem^10^ and our study also found it was differentially expressed in normal wood, tension wood, and opposite wood. We suspect that genetic variants in *Pt-miR397a* and its target genes may functionally interact with each other in determining metabolic responses. Our analysis identified the importance of epistasis as a principal factor that determines variation for quantitative traits and provides a means to uncover genetic networks affecting these traits. Knowledge of epistatic networks will contribute to our understanding of the genetic basis of evolutionarily and clinically important traits and enhance predictive ability at an individual level in medicine and agriculture^37^. However, the precise mechanisms underlying such gene-gene interactions are largely unknown. Further studies, especially experimental approaches, are needed to explore this hypothesis. In conclusion, in agreement with our hypothesis, association genetics can be useful for the exploration of function and interactions of miRNAs with their target genes.

## Methods

### Population and phenotype

An association population of 261 unrelated individuals, representing almost the entire climatic range and original provenance of *P. tomentosa*, were used for SNP association studies^14^. These trees were grown from 1,047 randomly sampled individuals assembled from an area of 1 million km^2^ along the Yellow River (30–40°N, 105–125°E), and were grown in Guan Xian County, Shandong Province, China (36°23′N, 115°47′E) using a randomized complete block design with three clonal replications. Nine quantitative traits were measured in the 261 individuals; the tree growth traits were diameter at breast height (DBH), tree height (H), and stem volume (V), and the wood property traits were holocellulose content, α-cellulose content, lignin content, fiber length, fiber width, and microfiber angle. The measurements of these phenotypic data were described in detail by Du *et al.*^38^.

### Gene extraction, SNP identification and genotyping

The full-length sequence of the *Pt-miR397a* gene was cloned as described by Lu *et al.*^16^. Identification of Pt-miR397a potential target genes was carried out by psRNATarget server^39^ (http://plantgrn.noble.org/psRNATarget/). Six potential target genes that have high abundantly and specific expression in xylem and also have unique sequences around the miRNA target sites were cloned, as described in our previous study^31^. To find SNPs of *Pt-miR397a* and its six target genes, these genes were sequenced from 40 individuals randomly selected from the association population, as described in our previous study^31^. All primers are provided in Table S5. The common SNPs (minor allele frequency, MAF > 5%, Table S6) were genotyped in 261 individuals of the association population using the method described by Du *et al.*^14^. To compare whether the SNP could affect the accessibility of mRNA target site to miRNA, target accessibility was calculated by psRNATarget, which employed RNAup and the energy that is required to open secondary structure around target site (ΔΔG)^39^.

### Measurement of gene expression by reverse transcription quantitative PCR (RT-qPCR)

RT-qPCR was performed on a 7500 Fast Real-Time PCR System using the SYBR Premix Ex Taq according to our previous study^40^. The cDNA template for the reactions was reverse-transcribed using total RNA extracted from cambium, developing xylem, mature leaf, and mature xylem from normal wood, opposite wood, and tension wood. Primer Express 3.0 software (Applied Biosystems) was used to design the primers for target genes. Poplar *Actin* (Accession number: EF145577) was used as the internal control for gene expression measurements. The relative level of *Pt-miR397a* was measured and standardized to 5.8S rRNA as described by Song *et al.*^40^. Triplicate technical and triplicate biological repetitions were performed on all reactions.

### Data analysis

The generalized linear model (GLM) in the software package TASSEL 2.1^41^ (http://www.maizegenetics.net/) was used to identify single-SNP associations in the association population. For multiple-SNP association, the fGWAS Package (Functional Genome-wide Association Studies) for simultaneous analysis and genetic modeling of multiple SNPs was used to identify and estimate the possible additive and dominant effects associated with all significant SNPs for each trait, and was run in R (http://www.r-project.org/). This package analyzes the genotypic and phenotypic data through a preconditioning step and a Bayesian Lasso model, and then summarizes the significant SNPs^27^. Multifactor Dimensionality Reduction 3.0.2 (MDR3.0.2) was applied to investigate epistatic interactions in our study^21^. The MDR algorithm detects interactions by combining attribute selection, attribute construction, classification, and permutation testing. The information gain (IG) was calculated by entropy-based measure to evaluate the genetic effects of significant SNP-SNP interactions.

The ratio of dominant (d) to additive (a) effects was used to quantify the modes of gene action. When the ratio of d to a effects was 0.50 < |d/a| < 1.25, gene action was defined as partial or complete dominance. Ratios of d to a effects in the range |d/a| ≤ 0.5 were equated with additive effects and ratios of |d/a| > 1.25 were defined as under- or overdominance^42^.

## Additional Information

**How to cite this article**: Chen, J. *et al.* Association genetics in *Populus* reveals the interactions between Pt-miR397a and its target genes. *Sci. Rep.* **5**, 11672; doi: 10.1038/srep11672 (2015).

**Accession codes**: The sequences were uploaded in NCBI GenBank (http://www.ncbi.nlm.nih.gov/nuccore/) with accession number KP403489-KP403528 (*Pt-miR397a*), KP403329-KP403368 (*Pt-LAC13*), 20 KP403329-KP403368 (*Pt-LAC18*), KP403369-KP403408 (*Pt-LAC20*), KP403289-KP403328 21 (*Pt-HSP40*), KP403449-KP403488 (*Pt-LEA*) and KP403529-KP403568 (*Pt-SPRY*).

## Supplementary Material

Supplementary Information

## Figures and Tables

**Table 1:** **The SNPs identified in Pt-miR397a and its target genes (Partial sequence around target sites).**

**Gene**	**Length (bp)**	**Number of Polymorphic Sites**	**Frequency (bp**^**−1**^)	**Gene model**
*Pt-miR397a*				
Mature region	21	0	0	
Premature region	120	3	40	
Other region	1147	54	21	
Total	1247	57	22	
*Pt-LAC13*	520	19	27	POPTR_0006s09520
*Pt-LAC18*	406	11	41	POPTR_0008s07370
*Pt-LAC20*	349	21	17	POPTR_0009s03940
*Pt-LEA*	824	31	25	POPTR_0001s18090
*Pt-HSP40*	414	11	38	POPTR_0007s06680
*Pt-SPRY*	519	14	37	POPTR_0019s11000

**Table 2:** **SNP markers significantly associated with growth and wood properties in the association population.**

**Trait**	**Locus**	**Genotype**	***P*** **value**	**R** ^**2**^ **(%)**
Lignin content	*Pt-LEA*-SNP29	A > G	0.001	11.86
Lignin content	*Pt-HSP40*-SNP01	C > A	0.006	8.66
Lignin content	*Pt-LEA*-SNP32	T > A	0.0033	12.57
Holocellulose content	*Pt-SPRY*-SNP08	T > C	0.0013	11.53
α-cellulose content	*Pt-miR397a*-SNP50	C > T	0.0032	9.90
α-cellulose content	*Pt-SPRY*-SNP08	T > C	0.0028	10.16
α-cellulose content	*Pt-LEA*-SNP33	C > A	0.0098	10.66
Fiber length	*Pt-LEA*-SNP32	T > A	3.12E-04	17.15
Fiber length	*Pt-LEA*-SNP33	C > A	0.004	12.25
Fiber width	*Pt-LEA*-SNP32	T > A	0.006	11.37
Microfiber angle	*Pt-LAC20*-SNP15	A > G	0.0046	12.34
Microfiber angle	*Pt-LEA*-SNP02	A > T	0.0056	9.23
Tree diameter at breast height	*Pt-LAC13*-SNP17	A > G	0.0063	8.53
Tree diameter at breast height	*Pt-LAC18*-SNP01	A > G	0.0034	9.76
Tree diameter at breast height	*Pt-miR397a*-SNP35	A > G	0.0048	9.07
Tree diameter at breast height	*Pt-miR397a*-SNP46	A > G	0.0041	12.37
Tree diameter at breast height	*Pt-LAC13*-SNP12	A > T	0.0058	11.66
Tree diameter at breast height	*Pt-LAC13*-SNP18	A > T	0.0076	11.06
Tree diameter at breast height	*Pt-HSP40*-SNP05	G > T	0.0052	11.90
Tree diameter at breast height	*Pt-SPRY*-SNP11	C > G	2.20E-04	15.06
Tree diameter at breast height	*Pt-LAC13*-SNP01	C > T	0.0038	9.55
Tree diameter at breast height	*Pt-miR397a*-SNP32	C > T	0.0047	9.13
Tree diameter at breast height	*Pt-LAC13*-SNP13	T > C	7.29E-04	12.75
Tree diameter at breast height	*Pt-LAC18*-SNP04	T > C	0.0033	9.79
Tree diameter at breast height	*Pt-miR397a*-SNP38	T > C	0.0045	9.21
Tree diameter at breast height	*Pt-miR397a*-SNP33	T > G	0.0048	9.07
Tree height	*Pt-LAC13*-SNP18	A > T	1.22E-04	19.45
Tree height	*Pt-HSP40*-SNP05	G > T	1.69E-04	18.82
Tree height	*Pt-LAC13*-SNP13	T > C	0.0091	7.80
Stem volume	*Pt-LAC13*-SNP17	A > G	0.0065	8.35
Stem volume	*Pt-LAC18*-SNP01	A > G	0.0037	9.47
Stem volume	*Pt-miR397a*-SNP35	A > G	0.0045	9.06
Stem volume	*Pt-miR397a*-SNP46	A > G	2.39E-04	17.94
Stem volume	*Pt-LAC13*-SNP12	A > T	0.0043	12.15
Stem volume	*Pt-miR397a*-SNP18	A > T	0.007	8.43
Stem volume	*Pt-SPRY*-SNP11	C > G	9.30E-04	12.13
Stem volume	*Pt-LAC13*-SNP01	C > T	0.0031	9.83
Stem volume	*Pt-miR397a*-SNP32	C > T	0.0055	8.69
Stem volume	*Pt-LAC13*-SNP13	T > C	0.002	10.62
Stem volume	*Pt-LAC18-*SNP04	T > C	0.0046	9.05
Stem volume	*Pt-miR397a*-SNP38	T > C	0.0044	9.13
Stem volume	*Pt-miR397a*-SNP33	T > G	0.0045	9.06

P values represent the significant level for association (the significance is P ≤ 0.01), R^2^ percentage of the phenotypic variance explained.
