# Supplementary material for: Association genetics in Populus reveals the interactions between Pt-miR397a and its target genes
Source: Sci Rep. 2015 Jun 26;5:11672. doi: 10.1038/srep11672 (PMC4481775; doi:10.1038/srep11672)
Supplement: Supplementary Information [file srep11672-s1.pdf]

**Association genetics in *Populus* reveals the interactions between Pt-miR397a and its target genes**

Jinhui Chen[1,2], Beibei Chen[1,2], Xiaohui Yang[1,2], Jiaxing Tian[1,2], Qingzhang Du[1,2] and Deqiang Zhang[1,2*]

[1]National Engineering Laboratory for Tree Breeding, College of Biological Sciences and Technology, Beijing Forestry University, No. 35, Qinghua East Road, Beijing 100083, P. R. China

[2]Key Laboratory of Genetics and Breeding in Forest Trees and Ornamental Plants, Ministry of Education, College of Biological Sciences and Technology, Beijing Forestry University, No. 35, Qinghua East Road, Beijing 100083, P. R. China

*Corresponding Author: Deqiang Zhang, Email: DeqiangZhang@bjfu.edu.cn; Tel: +86-10-62336007;

Fax: +86-10-62336164

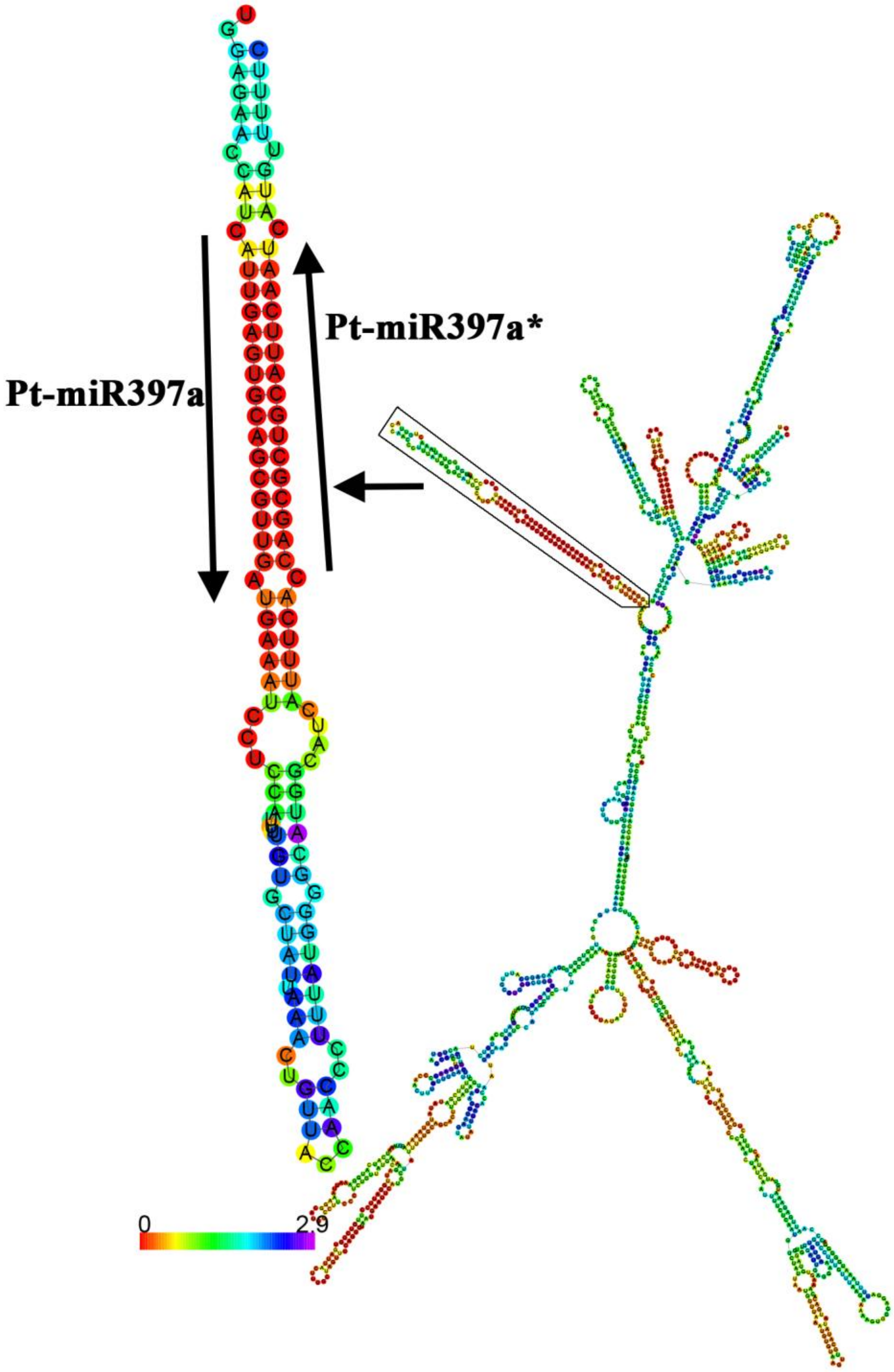


Figure S1 Prediction of the secondary structure of the *Pt-miR397a* transcript.

Table S1 Candidate target genes of Pt-miR397a identified using psRNATarget.

| **miRNA** | **Targets** | **Expectation** | **UPE** | **Inhibition** | **Target gene model** |
|---|---|---|---|---|---|
| Pt-**miR397a** | *Pt-LAC1* | 0.5 | 14.607 | Cleavage | POPTR_0001s14010.1 |
| Pt-**miR397a** | *Pt-LAC11* | 1.5 | 17.931 | Cleavage | POPTR_0006s08740.1 |
| Pt-**miR397a** | *Pt-LAC12* | 2.5 | 12.911 | Cleavage | POPTR_0006s08780.1 |
| Pt-**miR397a** | *Pt-LAC13* | 1.5 | 12.54 | Cleavage | POPTR_0006s09520.1 |
| Pt-**miR397a** | *Pt-LAC14* | 0.5 | 17.542 | Cleavage | POPTR_0006s09830.1 |
| Pt-**miR397a** | *Pt-LAC15* | 0.5 | 17.061 | Cleavage | POPTR_0006s09840.1 |
| Pt-**miR397a** | *Pt-LAC16* | 3 | 13.76 | Translation | POPTR_0007s13050.1 |
| Pt-**miR397a** | *Pt-LAC18* | 2 | 10.999 | Cleavage | POPTR_0008s07370.1 |
| Pt-**miR397a** | *Pt-LAC2* | 0.5 | 15.163 | Cleavage | POPTR_0001s18500.1 |
| Pt-**miR397a** | *Pt-LAC20* | 2 | 21.192 | Cleavage | POPTR_0009s03940.1 |
| Pt-**miR397a** | *Pt-LAC21* | 1.5 | 12.887 | Cleavage | POPTR_0009s04720.1 |
| Pt-**miR397a** | *Pt-LAC22* | 2 | 13.113 | Cleavage | POPTR_0009s10550.1 |
| Pt-**miR397a** | *Pt-LAC23* | 0.5 | 17.056 | Cleavage | POPTR_0009s15840.1 |
| Pt-**miR397a** | *Pt-LAC24* | 0.5 | 17.068 | Cleavage | POPTR_0009s15860.1 |
| Pt-**miR397a** | *Pt-LAC26* | 2 | 9.254 | Cleavage | POPTR_0010s19090.1 |
| Pt-**miR397a** | *Pt-LAC29* | 1.5 | 13.189 | Cleavage | POPTR_0011s12090.1 |
| Pt-**miR397a** | *Pt-LAC30* | 1.5 | 13.875 | Translation | POPTR_0011s12100.1 |
| Pt-**miR397a** | *Pt-LAC37* | 1.5 | 11.951 | Cleavage | POPTR_0016s11500.1 |
| Pt-**miR397a** | *Pt-LAC38* | 3 | 11.334 | Translation | POPTR_0016s11520.1 |
| Pt-**miR397a** | *Pt-LAC39* | 3 | 12.783 | Translation | POPTR_0016s11540.1 |
| Pt-**miR397a** | *Pt-LAC4* | 1.5 | 12.573 | Cleavage | POPTR_0001s25580.1 |
| Pt-**miR397a** | *Pt-LAC40* | 0.5 | 20.6 | Cleavage | POPTR_0016s11950.1 |
| Pt-**miR397a** | *Pt-LAC41* | 2 | 19.726 | Cleavage | POPTR_0016s11960.1 |
| Pt-**miR397a** | *Pt-LAC48* | 3 | 13.129 | Translation | POPTR_0091s00270.1 |
| Pt-**miR397a** | *Pt-LAC49* | 0.5 | 17.061 | Cleavage | POPTR_0958s00200.1 |
| Pt-**miR397a** | *Pt-LAC5* | 2.5 | 16.772 | Cleavage | POPTR_0001s35740.1 |
| Pt-**miR397a** | *Pt-LAC6* | 1.5 | 17.535 | Cleavage | POPTR_0001s41160.1 |
| Pt-**miR397a** | *Pt-LAC7* | 1 | 14.365 | Cleavage | POPTR_0001s41170.1 |
| Pt-**miR397a** | *Pt-LAC8* | 2 | 12.678 | Cleavage | POPTR_0004s16370.1 |
| Pt-**miR397a** | *Pt-LEA* | 2.5 | 11.636 | Cleavage | POPTR_0001s18090.1 |
| Pt-**miR397a** | *Pt-Hsp40* | 3 | 13.439 | Cleavage | POPTR_0007s06680.1 |
| Pt-**miR397a** | *Pt-SPRY* | 3 | 16.291 | Cleavage | POPTR_0019s11000.2 |
| Pt-**miR397a** | *Pt-SPRY* | 3 | 16.291 | Cleavage | POPTR_0019s11000.1 |
| Pt-**miR397a** | unknown | 3 | 22.938 | Cleavage | POPTR_0005s23060.1 |
| Pt-**miR397a** | unknown | 2 | 18.873 | Translation | POPTR_0001s05730.1 |
| Pt-**miR397a** | unknown | 3 | 21.164 | Cleavage | POPTR_0014s10160.1 |
| Pt-**miR397a** | unknown | 3 | 15.948 | Translation | POPTR_0005s21780.1 |
| Pt-**miR397a** | unknown | 3 | 15.948 | Translation | POPTR_0005s21780.2 |
| Pt-**miR397a** | unknown | 3 | 22.538 | Translation | POPTR_0018s02800.1 |
| Pt-**miR397a** | unknown | 3 | 9.954 | Cleavage | POPTR_0006s07740.1 |
| Pt-**miR397a** | unknown | 2 | 22.574 | Cleavage | POPTR_0001s41155.1 |

**Table S2 The additive and dominant effects calculated by multiple SNP-based associations of SNP across growth and wood property traits.**

| Trait | Locus | Additive Effect | Dominance Effect | \|d/a\| | $R^2$ (%) |
|---|---|---|---|---|---|
| stem volume | Pt-LAC13-SNP13 | 53.321 | 53.039 | 0.994711 | 14.397 |
| stem volume | Pt-LAC20-SNP9 | 11.823 | 30.069 | 2.543263 | 15.063 |
| stem volume | Pt-LEA-SNP8 | -10.545 | -21.438 | 2.033001 | 2.352 |
| stem volume | Pt-miR397a-SNP48 | 52.228 | 79.48 | 1.521789 | 11.393 |
| stem volume | Pt-miR397a-SNP50 | 6.421 | -21.932 | 3.415667 | 2.211 |
| stem volume | Pt-SPRY-SNP13 | 10.441 | 48.028 | 4.599943 | 5.284 |
| tree diameter at breast height | Pt-Hsp40-SNP10 | 1.732 | -0.015 | 0.008661 | 1.625 |
| tree diameter at breast height | Pt-LAC13-SNP13 | -9.374 | 13.506 | 1.440794 | 22.184 |
| tree diameter at breast height | Pt-LAC13-SNP18 | -4.561 | 1.374 | 0.30125 | 13.152 |
| tree diameter at breast height | Pt-LAC18-SNP07 | -0.879 | 0.525 | 0.59727 | 1.063 |
| tree diameter at breast height | Pt-miR397a-SNP14 | 0.533 | 1 | 1.876173 | 5.508 |
| tree diameter at breast height | Pt-miR397a-SNP16 | 1.767 | -2.947 | 1.667799 | 7.434 |
| tree diameter at breast height | Pt-miR397a-SNP34 | -1.488 | -5.273 | 3.543683 | 18.469 |
| tree diameter at breast height | Pt-miR397a-SNP48 | 6.276 | 3.01 | 0.479605 | 20.614 |
| tree diameter at breast height | Pt-miR397a-SNP5 | -0.75 | -0.111 | 0.148 | 1.527 |
| tree diameter at breast height | Pt-miR397a-SNP55 | -0.404 | -0.632 | 1.564356 | 1.96 |
| tree diameter at breast height | Pt-miR397a-SNP56 | 0.865 | -2.784 | 3.218497 | 4.896 |
| tree diameter at breast height | Pt-miR397a-SNP57 | -1.056 | 0.115 | 0.108902 | 2.208 |
| tree diameter at breast height | Pt-SPRY-SNP13 | 0.272 | 2.207 | 8.113971 | 3.542 |
| tree height | Pt-Hsp40-SNP10 | 3.58 | -1.172 | 0.327374 | 1.062 |
| tree height | Pt-Hsp40-SNP2 | 2.932 | 2.5 | 0.85266 | 16.309 |
| tree height | Pt-LAC18-SNP04 | -2.616 | 0.178 | 0.068043 | 5.806 |
| tree height | Pt-LAC20-SNP12 | -0.46 | 1.877 | 4.080435 | 5.647 |
| tree height | Pt-LAC20-SNP18 | -0.43 | 1.308 | 3.04186 | 1.275 |
| tree height | Pt-LAC20-SNP7 | -2.759 | 2.763 | 1.00145 | 20.757 |
| tree height | Pt-LEA-SNP4 | -0.024 | -1.243 | 51.79167 | 2.339 |
| tree height | Pt-LEA-SNP5 | 0.487 | 4.025 | 8.264887 | 15.722 |
| tree height | Pt-SPRY-SNP13 | 4.021 | 2.751 | 0.684158 | 7.21 |
| alpha-cellulose content | Pt-LAC18-SNP07 | 3.431 | -1.919 | 0.559312 | 2.04 |
| alpha-cellulose content | Pt-LAC20-SNP5 | 2.829 | 1.858 | 0.656769 | 6.6 |
| alpha-cellulose content | Pt-LEA-SNP17 | -5.587 | -1.733 | 0.310184 | 6.835 |
| alpha-cellulose content | Pt-LEA-SNP18 | -8.625 | 0.169 | 0.019594 | 22.547 |
| alpha-cellulose content | Pt-LEA-SNP19 | 6.902 | -0.864 | 0.125181 | 17.163 |
| alpha-cellulose content | Pt-LEA-SNP28 | -5.373 | 1.176 | 0.218872 | 2.666 |
| alpha-cellulose content | Pt-LEA-SNP3 | -0.324 | 1.251 | 3.861111 | 0.921 |
| alpha-cellulose content | Pt-LEA-SNP5 | -3.351 | 0.444 | 0.132498 | 2.761 |
| alpha-cellulose content | Pt-miR397a-SNP52 | 3.152 | -5.627 | 1.785216 | 3.536 |
| alpha-cellulose content | Pt-miR397a-SNP57 | 4.266 | 2.788 | 0.65354 | 12.243 |
| alpha-cellulose content | Pt-SPRY-SNP4 | 2.356 | 0.969 | 0.41129 | 2.095 |
| holocellulose content | Pt-LAC13-SNP18 | -5.985 | 8.869 | 1.481871 | 2.624 |
| holocellulose content | Pt-LEA-SNP17 | -8.211 | 5.531 | 0.673609 | 1.865 |
| holocellulose content | Pt-LEA-SNP28 | -3.798 | 12.556 | 3.305951 | 8.173 |
| holocellulose content | Pt-LEA-SNP6 | 2.907 | 3.601 | 1.238734 | 5.057 |
| holocellulose content | Pt-miR397a-SNP31 | -0.185 | -4.924 | 26.61622 | 1.178 |
| lignin content | Pt-LAC20-SNP10 | 0.78 | 1.612 | 2.066667 | 15.274 |
| lignin content | Pt-LAC20-SNP13 | -0.427 | 1.876 | 4.393443 | 13.764 |
| lignin content | Pt-LEA-SNP27 | -0.998 | 3.995 | 4.003006 | 10.043 |
| lignin content | Pt-LEA-SNP30 | -1.796 | 0.227 | 0.126392 | 4.459 |

| | | | | | |
|---|---|---|---|---|---|
| lignin content | Pt-LEA-SNP5 | -0.116 | -0.378 | 3.258621 | 0.518 |
| lignin content | Pt-miR397a-SNP14 | -0.837 | -0.438 | 0.523297 | 3.059 |
| lignin content | Pt-miR397a-SNP21 | 0.988 | 2.099 | 2.124494 | 13.464 |
| lignin content | Pt-miR397a-SNP4 | -0.086 | -0.561 | 6.523256 | 1.043 |
| lignin content | Pt-miR397a-SNP56 | -1.167 | 0.447 | 0.383033 | 0.57 |
| lignin content | Pt-SPRY-SNP12 | -3.52 | -1.068 | 0.303409 | 19.093 |
| fiber length | Pt-Hsp40-SNP9 | 0.061 | 0.036 | 0.590164 | 3.547 |
| fiber length | Pt-LAC13-SNP9 | -0.194 | -0.146 | 0.752577 | 13.843 |
| fiber length | Pt-LAC20-SNP10 | 0.035 | 0.045 | 1.285714 | 6.345 |
| fiber length | Pt-LAC20-SNP11 | 0.022 | -0.02 | 0.909091 | 1.222 |
| fiber length | Pt-LAC20-SNP8 | 0.007 | 0.045 | 6.428571 | 4.1 |
| fiber length | Pt-LEA-SNP30 | -0.094 | 0.177 | 1.882979 | 16.906 |
| fiber length | Pt-LEA-SNP31 | -0.01 | -0.029 | 2.9 | 1.309 |
| fiber length | Pt-LEA-SNP6 | -0.039 | -0.048 | 1.230769 | 5.067 |
| fiber length | Pt-miR397a-SNP16 | -0.094 | 0.034 | 0.361702 | 2.726 |
| fiber length | Pt-miR397a-SNP20 | -0.029 | 0.004 | 0.137931 | 0.884 |
| fiber length | Pt-miR397a-SNP30 | 0.452 | -0.536 | 1.185841 | 7.667 |
| fiber length | Pt-miR397a-SNP48 | 0.121 | -0.009 | 0.07438 | 1.153 |
| fiber length | Pt-miR397a-SNP55 | -0.031 | 0.059 | 1.903226 | 2.133 |
| fiber length | Pt-miR397a-SNP56 | -0.068 | -0.058 | 0.852941 | 5.218 |
| fiber length | Pt-miR397a-SNP57 | -0.003 | -0.048 | 16 | 2.717 |
| fiber length | Pt-miR397a-SNP7 | 0.063 | -0.093 | 1.47619 | 2.965 |
| fiber length | Pt-SPRY-SNP11 | -0.075 | -0.032 | 0.426667 | 5.832 |
| fiber length | Pt-SPRY-SNP13 | -0.041 | -0.067 | 1.634146 | 2.387 |
| fiber width | Pt-Hsp40-SNP2 | -1.135 | 2.914 | 2.567401 | 14.91 |
| fiber width | Pt-LEA-SNP26 | 1.015 | -0.952 | 0.937931 | 5.026 |
| fiber width | Pt-LEA-SNP30 | -2.058 | 1.18 | 0.573372 | 3.993 |
| fiber width | Pt-miR397a-SNP13 | -0.668 | 0.42 | 0.628743 | 1.636 |
| fiber width | Pt-miR397a-SNP14 | -0.325 | -0.972 | 2.990769 | 3.66 |
| fiber width | Pt-miR397a-SNP3 | 0.208 | 0.383 | 1.841346 | 0.745 |
| fiber width | Pt-miR397a-SNP5 | -0.094 | -0.404 | 4.297872 | 0.518 |
| fiber width | Pt-miR397a-SNP7 | 1.217 | -1.384 | 1.137223 | 1.478 |
| fiber width | Pt-SPRY-SNP12 | 1.287 | 3.372 | 2.620047 | 19.843 |
| microfibril angle | Pt-Hsp40-SNP9 | -3.945 | 5.756 | 1.459062 | 9.302 |
| microfibril angle | Pt-LAC20-SNP3 | 0.365 | 0.434 | 1.189041 | 0.893 |
| microfibril angle | Pt-LEA-SNP4 | 1.939 | 1.578 | 0.813822 | 16.75 |
| microfibril angle | Pt-LEA-SNP6 | 0.667 | -0.852 | 1.277361 | 0.653 |
| microfibril angle | Pt-miR397a-SNP14 | -1.151 | 1.072 | 0.931364 | 2.134 |
| microfibril angle | Pt-miR397a-SNP3 | -0.092 | 1.096 | 11.91304 | 2.396 |
| microfibril angle | Pt-miR397a-SNP5 | -0.464 | -1.535 | 3.30819 | 5.333 |
| microfibril angle | Pt-miR397a-SNP6 | -1.576 | -1.397 | 0.886421 | 12.23 |

**Table S3 Main effects detected by Multifactor Dimensionality Reduction 3.0.2 under epistasis gene model**

| Attribute | H(A) | H(A\|C) | I(A;C) | Associated traits |
|---|---|---|---|---|
| Pt-miR397a-SNP36 | 0.4798 | 0.479 | 0.0008 | lignin content |
| Pt-miR397a-SNP32 | 0.4038 | 0.4027 | 0.0011 | lignin content |
| Pt-Hsp40-SNP01 | 0.8856 | 0.8838 | 0.0019 | lignin content |
| Pt-Hsp40-SNP01 | 0.8856 | 0.8838 | 0.0019 | holocellulose content |
| Pt-LAC20-SNP21 | 0.3173 | 0.3153 | 0.002 | alpha-cellulose content |
| Pt-SPRY-SNP01 | 0.4732 | 0.4713 | 0.002 | fiber length |
| Pt-miR397a-SNP51 | 0.7355 | 0.7327 | 0.0028 | microfibril angle |
| Pt-SPRY-SNP12 | 0.4038 | 0.3925 | 0.0113 | tree diameter at breast height |
| Pt-miR397a-SNP34 | 0.2481 | 0.2367 | 0.0114 | fiber length |
| Pt-SPRY-SNP03 | 0.4514 | 0.4396 | 0.0118 | lignin content |
| Pt-miR397a-SNP34 | 0.2481 | 0.2363 | 0.0118 | alpha-cellulose content |
| Pt-miR397a-SNP34 | 0.2481 | 0.2363 | 0.0118 | tree diameter at breast height |
| Pt-LEA-SNP30 | 0.7317 | 0.7195 | 0.0122 | alpha-cellulose content |
| Pt-LEA-SNP30 | 0.7317 | 0.7195 | 0.0122 | fiber width |
| Pt-miR397a-SNP03 | 1.3662 | 1.353 | 0.0132 | holocellulose content |
| Pt-SPRY-SNP02 | 0.4069 | 0.3937 | 0.0132 | fiber length |
| Pt-LEA-SNP08 | 0.4069 | 0.3937 | 0.0132 | tree height |
| Pt-LAC13-SNP18 | 0.6262 | 0.6119 | 0.0143 | tree diameter at breast height |
| Pt-Hsp40-SNP10 | 0.3064 | 0.292 | 0.0143 | stem volume |
| Pt-Hsp40-SNP11 | 0.8498 | 0.834 | 0.0158 | holocellulose content |
| Pt-Hsp40-SNP06 | 1.0616 | 1.0458 | 0.0158 | stem volume |
| Pt-LAC13-SNP04 | 0.8158 | 0.799 | 0.0168 | holocellulose content |
| Pt-miR397a-SNP50 | 0.6084 | 0.5881 | 0.0203 | tree diameter at breast height |
| Pt-LAC20-SNP12 | 1.7695 | 1.7487 | 0.0209 | tree height |
| Pt-LEA-SNP09 | 0.7778 | 0.7567 | 0.0211 | fiber width |
| Pt-miR397a-SNP46 | 0.4929 | 0.4696 | 0.0234 | alpha-cellulose content |
| Pt-miR397a-SNP48 | 0.1809 | 0.1571 | 0.0238 | alpha-cellulose content |
| Pt-LAC20-SNP19 | 0.7317 | 0.7059 | 0.0258 | stem volume |
| Pt-LAC20-SNP19 | 0.7317 | 0.705 | 0.0267 | lignin content |
| Pt-SPRY-SNP04 | 0.7239 | 0.6942 | 0.0298 | microfibril angle |
| Pt-miR397a-SNP49 | 0.2481 | 0.2137 | 0.0344 | tree height |
| Pt-miR397a-SNP23 | 0.5819 | 0.5463 | 0.0356 | tree height |
| Pt-miR397a-SNP07 | 0.9731 | 0.9349 | 0.0382 | tree height |
| Pt-miR397a-SNP03 | 1.3662 | 1.3264 | 0.0399 | alpha-cellulose content |
| Pt-miR397a-SNP07 | 0.9731 | 0.9306 | 0.0425 | tree diameter at breast height |
| Pt-LEA-SNP04 | 1.2521 | 1.2013 | 0.0508 | stem volume |
| Pt-miR397a-SNP31 | 0.3731 | 0.3141 | 0.059 | fiber length |
| Pt-miR397a-SNP37 | 0.3621 | 0.2882 | 0.0739 | holocellulose content |
| Pt-SPRY-SNP11 | 0.8799 | 0.7831 | 0.0967 | tree height |

**Table S4 Associations detected by Multifactor Dimensionality Reduction 3.0.2 under epistasis gene model**

| Attribute A | Attribute B | Pairwise epistaisis effect | Effect of interaction | Associated traits |
|---|---|---|---|---|
| Pt-Hsp40-SNP01 | Pt-SPRY-SNP03 | -0.0089 | 0.0048 | lignin content |
| Pt-Hsp40-SNP01 | Pt-SPRY-SNP08 | 0.0068 | 0.0087 | lignin content |
| Pt-Hsp40-SNP10 | Pt-Hsp40-SNP06 | -0.0172 | 0.0129 | stem volume |
| Pt-Hsp40-SNP11 | Pt-Hsp40-SNP01 | -0.0063 | 0.0113 | holocellulose content |
| Pt-Hsp40-SNP11 | Pt-SPRY-SNP04 | -0.027 | 0.0028 | microfibril angle |
| Pt-LAC13-SNP04 | Pt-Hsp40-SNP11 | -0.031 | 0.0016 | holocellulose content |
| Pt-LAC13-SNP09 | Pt-Hsp40-SNP06 | -0.0057 | 0.0101 | stem volume |
| Pt-LAC13-SNP09 | Pt-Hsp40-SNP10 | -0.0123 | 0.002 | stem volume |
| Pt-LAC13-SNP09 | Pt-LAC20-SNP19 | -0.0234 | 0.0024 | stem volume |
| Pt-LAC13-SNP09 | Pt-LEA-SNP04 | -0.0393 | 0.0115 | stem volume |
| Pt-LAC13-SNP18 | Pt-SPRY-SNP08 | -0.0123 | 0.0021 | tree diameter at breast height |
| Pt-LAC13-SNP18 | Pt-SPRY-SNP12 | -0.0045 | 0.0212 | tree diameter at breast height |
| Pt-LAC20-SNP12 | Pt-LEA-SNP08 | -0.0091 | 0.025 | tree height |
| Pt-LAC20-SNP12 | Pt-SPRY-SNP11 | 0.0123 | 0.1299 | tree height |
| Pt-LAC20-SNP19 | Pt-Hsp40-SNP01 | -0.0209 | 0.0077 | lignin content |
| Pt-LAC20-SNP19 | Pt-Hsp40-SNP06 | -0.0339 | 0.0077 | stem volume |
| Pt-LAC20-SNP19 | Pt-Hsp40-SNP10 | -0.0377 | 0.0024 | stem volume |
| Pt-LAC20-SNP19 | Pt-LEA-SNP04 | -0.0687 | 0.0079 | stem volume |
| Pt-LAC20-SNP19 | Pt-SPRY-SNP03 | -0.0155 | 0.023 | lignin content |
| Pt-LAC20-SNP19 | Pt-SPRY-SNP08 | -0.0252 | 0.0015 | lignin content |
| Pt-LAC20-SNP21 | Pt-LEA-SNP30 | 0.007 | 0.0212 | alpha-cellulose content |
| Pt-LEA-SNP04 | Pt-Hsp40-SNP06 | -0.0443 | 0.0223 | stem volume |
| Pt-LEA-SNP04 | Pt-Hsp40-SNP10 | -0.0167 | 0.0484 | stem volume |
| Pt-LEA-SNP08 | Pt-SPRY-SNP11 | -0.0132 | 0.0967 | tree height |
| Pt-LEA-SNP09 | Pt-Hsp40-SNP09 | -0.0012 | 0.0199 | fiber width |
| Pt-LEA-SNP30 | Pt-Hsp40-SNP01 | 0.0695 | 0.0714 | holocellulose content |
| Pt-LEA-SNP30 | Pt-Hsp40-SNP11 | 0.019 | 0.0348 | holocellulose content |
| Pt-LEA-SNP30 | Pt-LEA-SNP09 | -0.0121 | 0.0212 | fiber width |
| Pt-LEA-SNP30 | Pt-SPRY-SNP04 | -0.0036 | 0.0262 | microfibril angle |
| Pt-miR397a-SNP03 | Pt-Hsp40-SNP01 | -0.0108 | 0.0042 | holocellulose content |
| Pt-miR397a-SNP03 | Pt-Hsp40-SNP11 | -0.0261 | 0.0028 | holocellulose content |
| Pt-miR397a-SNP03 | Pt-LAC13-SNP04 | -0.0242 | 0.0058 | holocellulose content |
| Pt-miR397a-SNP03 | Pt-LAC20-SNP21 | -0.0169 | 0.025 | alpha-cellulose content |
| Pt-miR397a-SNP03 | Pt-LEA-SNP30 | 0.0098 | 0.023 | holocellulose content |
| Pt-miR397a-SNP03 | Pt-LEA-SNP30 | -0.0291 | 0.023 | alpha-cellulose content |
| Pt-miR397a-SNP07 | Pt-LAC13-SNP18 | -0.048 | 0.0088 | tree diameter at breast height |
| Pt-miR397a-SNP07 | Pt-LAC20-SNP12 | 0.0014 | 0.0604 | tree height |
| Pt-miR397a-SNP07 | Pt-LEA-SNP08 | -0.0154 | 0.036 | tree height |
| Pt-miR397a-SNP07 | Pt-SPRY-SNP08 | -0.0258 | 0.0167 | tree diameter at breast height |
| Pt-miR397a-SNP07 | Pt-SPRY-SNP11 | -0.0382 | 0.0967 | tree height |
| Pt-miR397a-SNP07 | Pt-SPRY-SNP12 | -0.0416 | 0.0122 | tree diameter at breast height |
| Pt-miR397a-SNP23 | Pt-LAC20-SNP12 | -0.0179 | 0.0385 | tree height |
| Pt-miR397a-SNP23 | Pt-LEA-SNP08 | -0.0207 | 0.0281 | tree height |
| Pt-miR397a-SNP23 | Pt-miR397a-SNP07 | -0.0367 | 0.0372 | tree height |
| Pt-miR397a-SNP23 | Pt-SPRY-SNP11 | 0.0085 | 0.1408 | tree height |
| Pt-miR397a-SNP31 | Pt-SPRY-SNP01 | -0.0266 | 0.0344 | fiber length |
| Pt-miR397a-SNP31 | Pt-SPRY-SNP02 | -0.0007 | 0.0714 | fiber length |
| Pt-miR397a-SNP32 | Pt-Hsp40-SNP01 | -0.0011 | 0.0019 | lignin content |

| | | | | |
|---|---|---|---|---|
| Pt-miR397a-SNP32 | Pt-LAC20-SNP19 | -0.0048 | 0.023 | lignin content |
| Pt-miR397a-SNP32 | Pt-SPRY-SNP03 | 0.0109 | 0.0238 | lignin content |
| Pt-miR397a-SNP32 | Pt-SPRY-SNP08 | 0.0029 | 0.004 | lignin content |
| Pt-miR397a-SNP34 | Pt-LAC13-SNP18 | -0.0098 | 0.0163 | tree diameter at breast height |
| Pt-miR397a-SNP34 | Pt-LAC20-SNP21 | -0.0073 | 0.0065 | alpha-cellulose content |
| Pt-miR397a-SNP34 | Pt-LEA-SNP30 | 0.0047 | 0.0287 | alpha-cellulose content |
| Pt-miR397a-SNP34 | Pt-miR397a-SNP03 | -0.0341 | 0.0176 | alpha-cellulose content |
| Pt-miR397a-SNP34 | Pt-miR397a-SNP07 | -0.0268 | 0.0275 | tree diameter at breast height |
| Pt-miR397a-SNP34 | Pt-miR397a-SNP31 | -0.0114 | 0.059 | fiber length |
| Pt-miR397a-SNP34 | Pt-SPRY-SNP01 | -0.0107 | 0.0027 | fiber length |
| Pt-miR397a-SNP34 | Pt-SPRY-SNP02 | -0.0016 | 0.023 | fiber length |
| Pt-miR397a-SNP34 | Pt-SPRY-SNP08 | -0.0063 | 0.0055 | tree diameter at breast height |
| Pt-miR397a-SNP34 | Pt-SPRY-SNP12 | 0.0006 | 0.0238 | tree diameter at breast height |
| Pt-miR397a-SNP36 | Pt-Hsp40-SNP01 | -0.0006 | 0.0021 | lignin content |
| Pt-miR397a-SNP36 | Pt-LAC20-SNP19 | -0.0045 | 0.023 | lignin content |
| Pt-miR397a-SNP37 | Pt-Hsp40-SNP01 | -0.071 | 0.0048 | holocellulose content |
| Pt-miR397a-SNP37 | Pt-Hsp40-SNP11 | -0.0363 | 0.0533 | holocellulose content |
| Pt-miR397a-SNP37 | Pt-LAC13-SNP04 | -0.0873 | 0.0034 | holocellulose content |
| Pt-miR397a-SNP46 | Pt-LAC20-SNP21 | -0.0114 | 0.0139 | alpha-cellulose content |
| Pt-miR397a-SNP46 | Pt-LEA-SNP30 | -0.0069 | 0.0287 | alpha-cellulose content |
| Pt-miR397a-SNP46 | Pt-miR397a-SNP34 | -0.0324 | 0.0027 | alpha-cellulose content |
| Pt-miR397a-SNP48 | Pt-LAC20-SNP21 | -0.0193 | 0.0065 | alpha-cellulose content |
| Pt-miR397a-SNP48 | Pt-LEA-SNP30 | -0.0148 | 0.0212 | alpha-cellulose content |
| Pt-miR397a-SNP48 | Pt-miR397a-SNP03 | -0.0396 | 0.024 | alpha-cellulose content |
| Pt-miR397a-SNP48 | Pt-miR397a-SNP34 | -0.0242 | 0.0114 | alpha-cellulose content |
| Pt-miR397a-SNP48 | Pt-miR397a-SNP46 | -0.0357 | 0.0114 | alpha-cellulose content |
| Pt-miR397a-SNP49 | Pt-LAC20-SNP12 | -0.0153 | 0.0399 | tree height |
| Pt-miR397a-SNP49 | Pt-LEA-SNP08 | -0.0246 | 0.023 | tree height |
| Pt-miR397a-SNP49 | Pt-miR397a-SNP07 | -0.0584 | 0.0142 | tree height |
| Pt-miR397a-SNP49 | Pt-miR397a-SNP23 | -0.0352 | 0.0348 | tree height |
| Pt-miR397a-SNP49 | Pt-SPRY-SNP11 | -0.0222 | 0.1089 | tree height |
| Pt-miR397a-SNP50 | Pt-LAC13-SNP18 | -0.005 | 0.0296 | tree diameter at breast height |
| Pt-miR397a-SNP50 | Pt-miR397a-SNP07 | -0.0273 | 0.0355 | tree diameter at breast height |
| Pt-miR397a-SNP50 | Pt-miR397a-SNP34 | -0.0046 | 0.0275 | tree diameter at breast height |
| Pt-miR397a-SNP50 | Pt-SPRY-SNP12 | -0.0113 | 0.0203 | tree diameter at breast height |
| Pt-miR397a-SNP51 | Pt-LEA-SNP09 | -0.0066 | 0.0145 | fiber width |
| Pt-miR397a-SNP51 | Pt-LEA-SNP30 | 0.0108 | 0.023 | fiber width |
| Pt-miR397a-SNP51 | Pt-LEA-SNP30 | 0.0201 | 0.023 | microfibril angle |
| Pt-miR397a-SNP51 | Pt-SPRY-SNP04 | -0.0263 | 0.0063 | microfibril angle |

**Table S5** Real-time PCR primer sequences

| **Gene/miRNA** | **Primer sequence** (5' > 3') |
|---|---|
| **Pt-SPRY** | Forward: GCGTGAGATTGTGGCTGATG |
| | Reverse: TCCTTCACGTTGGGATCTGTT |
| **Pt-Hsp40** | Forward: AGCTGCAACACTACTGGGTCTTC |
| | Reverse: TCCTGCCCCCAGTTGTTTTA |
| **Pt-LEA** | Forward: TGGTGCAGCATAAGCTAAAAGATC |
| | Reverse: GCCCAGGTTTTCCTGTCTATACA |
| **Pt-LAC20** | Forward: GGTTAAGGATGGCATGGATTGT |
| | Reverse: TGGAGGAGGGAGCTTCTGACT |
| **Pt-LAC18** | Forward: CAATCGGCCTAGGACTCAACA |
| | Reverse: GTCCTTGACACCGCCTAGCT |
| **ACTIN** | Forward: CTCCATCATGAAATGCGATG |
| | Reverse: TTGGGGCTAGTGCTGAGATT |
| **Pt-miR397a** | TCATTGAGTGCAGCGTTGATG |
| **Poly(T) adapter** | GCGAGCACAGAATTAATACGACTCACTATAGG(T)12VN* |
| **Reverse primer** | GCGAGCACAGAATTAATACGAC |
| **5.8S rRNA** | GTCTGCCTGGGTGTCACGCAA |

*V = A, G, C; N = A, T, G, C.

**Table S6** The SNPs identified in our study.

| ID | Location | Genotype |
|---|---|---|
| ***Pt-Hsp40*-SNP01** | Chr07-10216527 | C→A |
| ***Pt-Hsp40*-SNP02** | Chr07-10216542 | G→A |
| ***Pt-Hsp40*-SNP03** | Chr07-10216545 | A→G |
| ***Pt-Hsp40*-SNP04** | Chr07-10216547 | G→A,T |
| ***Pt-Hsp40*-SNP05** | Chr07-10216578 | C→A |
| ***Pt-Hsp40*-SNP06** | Chr07-10216586 | A→T |
| ***Pt-Hsp40*-SNP07** | Chr07-10216753 | G→T |
| ***Pt-Hsp40*-SNP08** | Chr07-10216754 | C→G |
| ***Pt-Hsp40*-SNP09** | Chr07-10216761 | T→A |
| ***Pt-Hsp40*-SNP10** | Chr07-10216785 | C→G,T |
| ***Pt-Hsp40*-SNP11** | Chr07-10216894 | T→C |
| ***Pt-LAC13*-SNP01** | Chr06-7030293 | C→T |
| ***Pt-LAC13*-SNP02** | Chr06-7030304 | C→A |
| ***Pt-LAC13*-SNP03** | Chr06-7030307 | A→G |
| ***Pt-LAC13*-SNP04** | Chr06-7030336 | T→C |
| ***Pt-LAC13*-SNP05** | Chr06-7030339 | C→G |
| ***Pt-LAC13*-SNP06** | Chr06-7030406 | C→T |
| ***Pt-LAC13*-SNP07** | Chr06-7030427 | A→G |
| ***Pt-LAC13*-SNP08** | Chr06-7030467 | A→G |
| ***Pt-LAC13*-SNP09** | Chr06-7030539 | C→G |
| ***Pt-LAC13*-SNP10** | Chr06-7030542 | T→C |
| ***Pt-LAC13*-SNP11** | Chr06-7030658 | T→C |
| ***Pt-LAC13*-SNP12** | Chr06-7030686 | A→T |
| ***Pt-LAC13*-SNP13** | Chr06-7030695 | T→C |
| ***Pt-LAC13*-SNP14** | Chr06-7030705 | G→A |
| ***Pt-LAC13*-SNP15** | Chr06-7030707 | G→A |
| ***Pt-LAC13*-SNP16** | Chr06-7030709 | C→T |
| ***Pt-LAC13*-SNP17** | Chr06-7030735 | A→G |
| ***Pt-LAC13*-SNP18** | Chr06-7030752 | A→T |
| ***Pt-LAC13*-SNP19** | Chr06-7030758 | C→G |
| ***Pt-LAC18*-SNP01** | Chr08-4558929 | A→C |
| ***Pt-LAC18*-SNP02** | Chr08-4559034 | A→C |
| ***Pt-LAC18*-SNP03** | Chr08-4558634 | A→G |
| ***Pt-LAC18*-SNP04** | Chr08-4558679 | A→G |
| ***Pt-LAC18*-SNP05** | Chr08-4558948 | A→G |
| ***Pt-LAC18*-SNP06** | Chr08-4558821 | G→A |
| ***Pt-LAC18*-SNP07** | Chr08-4558830 | G→T |
| ***Pt-LAC18*-SNP08** | Chr08-4558933 | G→T |
| ***Pt-LAC18*-SNP09** | Chr08-4558918 | T→C |
| ***Pt-LAC18*-SNP10** | Chr08-4558952 | T→C |
| ***Pt-LAC18*-SNP11** | Chr08-4559020 | T→C |
| ***Pt-LAC20*-SNP01** | Chr01-16069392 | T→C |

| | | |
|---|---|---|
| ***Pt-LAC20*-SNP02** | Chr01-16069412 | C→T |
| ***Pt-LAC20*-SNP03** | Chr01-16069443 | G→A |
| ***Pt-LAC20*-SNP04** | Chr01-16069452 | A→G |
| ***Pt-LAC20*-SNP05** | Chr01-16069468 | G→A |
| ***Pt-LAC20*-SNP06** | Chr01-16069488 | C→T |
| ***Pt-LAC20*-SNP07** | Chr01-16069491 | G→C |
| ***Pt-LAC20*-SNP08** | Chr01-16069525 | A→G |
| ***Pt-LAC20*-SNP09** | Chr01-16069533 | G→A |
| ***Pt-LAC20*-SNP10** | Chr01-16069542 | A→T |
| ***Pt-LAC20*-SNP11** | Chr01-16069548 | T→C |
| ***Pt-LAC20*-SNP12** | Chr01-16069550 | T→C |
| ***Pt-LAC20*-SNP13** | Chr01-16069573 | C→T |
| ***Pt-LAC20*-SNP14** | Chr01-16069582 | A→G |
| ***Pt-LAC20*-SNP15** | Chr01-16069587 | A→G |
| ***Pt-LAC20*-SNP16** | Chr01-16069603 | A→G |
| ***Pt-LAC20*-SNP17** | Chr01-16069637 | C→T |
| ***Pt-LAC20*-SNP18** | Chr01-16069641 | A→G |
| ***Pt-LAC20*-SNP19** | Chr01-16069665 | T→C |
| ***Pt-LAC20*-SNP20** | Chr01-16069683 | A→G |
| ***Pt-LAC20*-SNP21** | Chr01-16069684 | T→A |
| ***Pt-LEA*-SNP01** | Chr01-15277904 | A→C |
| ***Pt-LEA*-SNP02** | Chr01-15277905 | A→T |
| ***Pt-LEA*-SNP03** | Chr01-15277910 | A→G |
| ***Pt-LEA*-SNP04** | Chr01-15277912 | G→A |
| ***Pt-LEA*-SNP05** | Chr01-15277926 | C→A |
| ***Pt-LEA*-SNP06** | Chr01-15277940 | G→T |
| ***Pt-LEA*-SNP07** | Chr01-15277963 | A→T |
| ***Pt-LEA*-SNP08** | Chr01-15277980 | T→G |
| ***Pt-LEA*-SNP09** | Chr01-15278065 | T→C |
| ***Pt-LEA*-SNP10** | Chr01-15278076 | C→T |
| ***Pt-LEA*-SNP11** | Chr01-15278181 | A→C |
| ***Pt-LEA*-SNP12** | Chr01-15278202 | T→A |
| ***Pt-LEA*-SNP13** | Chr01-15278284 | C→A |
| ***Pt-LEA*-SNP14** | Chr01-15278313 | T→C |
| ***Pt-LEA*-SNP15** | Chr01-15278322 | T→C |
| ***Pt-LEA*-SNP16** | Chr01-15278373 | T→C |
| ***Pt-LEA*-SNP17** | Chr01-15278420 | G→C |
| ***Pt-LEA*-SNP18** | Chr01-15278438 | T→A |
| ***Pt-LEA*-SNP19** | Chr01-15278454 | T→G |
| ***Pt-LEA*-SNP20** | Chr01-15278534 | A→G |
| ***Pt-LEA*-SNP21** | Chr01-15278540 | G→A |
| ***Pt-LEA*-SNP22** | Chr01-15278545 | G→C |
| ***Pt-LEA*-SNP23** | Chr01-15278570 | A→T |
| ***Pt-LEA*-SNP24** | Chr01-15278589 | A→C |

| | | |
|---|---|---|
| ***Pt-LEA*-SNP25** | Chr01-15278591 | C→A |
| ***Pt-LEA*-SNP26** | Chr01-15278601 | A→G |
| ***Pt-LEA*-SNP27** | Chr01-15278641 | T→C |
| ***Pt-LEA*-SNP28** | Chr01-15278666 | T→C |
| ***Pt-LEA*-SNP29** | Chr01-15278669 | A→G |
| ***Pt-LEA*-SNP30** | Chr01-15278675 | T→A |
| ***Pt-LEA*-SNP31** | Chr01-15278682 | C→A |
| ***Pt-miR397a*-SNP01** | Chr11-3440663 | A→C |
| ***Pt-miR397a*-SNP02** | Chr11-3440674 | G→T |
| ***Pt-miR397a*-SNP03** | Chr11-3440684 | T→C |
| ***Pt-miR397a*-SNP04** | Chr11-3440693 | C→A |
| ***Pt-miR397a*-SNP05** | Chr11-3440704 | A→G |
| ***Pt-miR397a*-SNP06** | Chr11-3440718 | T→G |
| ***Pt-miR397a*-SNP07** | Chr11-3440728 | G→T |
| ***Pt-miR397a*-SNP08** | Chr11-3440755 | G→A |
| ***Pt-miR397a*-SNP09** | Chr11-3440771 | T→G |
| ***Pt-miR397a*-SNP10** | Chr11-3440882 | G→C |
| ***Pt-miR397a*-SNP11** | Chr11-3440884 | A→T |
| ***Pt-miR397a*-SNP12** | Chr11-3440889 | A→T |
| ***Pt-miR397a*-SNP13** | Chr11-3440935 | G→C |
| ***Pt-miR397a*-SNP14** | Chr11-3440994 | T→C |
| ***Pt-miR397a*-SNP15** | Chr11-3441036 | G→T |
| ***Pt-miR397a*-SNP16** | Chr11-3441037 | G→T |
| ***Pt-miR397a*-SNP17** | Chr11-3441040 | T→C |
| ***Pt-miR397a*-SNP18** | Chr11-3441043 | A→T |
| ***Pt-miR397a*-SNP19** | Chr11-3441050 | A→G |
| ***Pt-miR397a*-SNP20** | Chr11-3441092 | C→T |
| ***Pt-miR397a*-SNP21** | Chr11-3441127 | A→G |
| ***Pt-miR397a*-SNP22** | Chr11-3441128 | A→G |
| ***Pt-miR397a*-SNP23** | Chr11-3441137 | A→C |
| ***Pt-miR397a*-SNP24** | Chr11-3441149 | A→C |
| ***Pt-miR397a*-SNP25** | Chr11-3441234 | C→T |
| ***Pt-miR397a*-SNP26** | Chr11-3441251 | T→A |
| ***Pt-miR397a*-SNP27** | Chr11-3441263 | G→T |
| ***Pt-miR397a*-SNP28** | Chr11-3441264 | A→T |
| ***Pt-miR397a*-SNP29** | Chr11-3441274 | A→G |
| ***Pt-miR397a*-SNP30** | Chr11-3441296 | G→C |
| ***Pt-miR397a*-SNP31** | Chr11-3441351 | A→G |
| ***Pt-miR397a*-SNP32** | Chr11-3441361 | C→T |
| ***Pt-miR397a*-SNP33** | Chr11-3441398 | T→G |
| ***Pt-miR397a*-SNP34** | Chr11-3441406 | T→G |
| ***Pt-miR397a*-SNP35** | Chr11-3441428 | A→G |
| ***Pt-miR397a*-SNP36** | Chr11-3441430 | A→C |
| ***Pt-miR397a*-SNP37** | Chr11-3441455 | A→G |

| | | |
|---|---|---|
| ***Pt-miR397a*-SNP38** | Chr11-3441460 | T→C |
| ***Pt-miR397a*-SNP39** | Chr11-3441473 | A→G |
| ***Pt-miR397a*-SNP40** | Chr11-3441575 | T→C |
| ***Pt-miR397a*-SNP41** | Chr11-3441608 | T→C |
| ***Pt-miR397a*-SNP42** | Chr11-3441610 | A→G |
| ***Pt-miR397a*-SNP43** | Chr11-3441722 | G→C |
| ***Pt-miR397a*-SNP44** | Chr11-3441752 | C→T |
| ***Pt-miR397a*-SNP45** | Chr11-3441807 | T→C |
| ***Pt-miR397a*-SNP46** | Chr11-3441810 | A→G |
| ***Pt-miR397a*-SNP47** | Chr11-3441820 | C→A |
| ***Pt-miR397a*-SNP48** | Chr11-3441835 | T→G |
| ***Pt-miR397a*-SNP49** | Chr11-3441856 | T→C |
| ***Pt-miR397a*-SNP50** | Chr11-3441875 | C→T |
| ***Pt-miR397a*-SNP51** | Chr11-3441877 | T→C |
| ***Pt-miR397a*-SNP52** | Chr11-3441878 | T→C |
| ***Pt-miR397a*-SNP53** | Chr11-3441903 | T→A |
| ***Pt-miR397a*-SNP54** | Chr11-3441922 | T→G |
| ***Pt-miR397a*-SNP55** | Chr11-3441947 | A→G |
| ***Pt-miR397a*-SNP56** | Chr11-3441999 | G→A |
| ***Pt-miR397a*-SNP57** | Chr11-3442042 | A→G |
| ***Pt-SPRY*-SNP01** | Chr19-11405509 | G→A |
| ***Pt-SPRY*-SNP02** | Chr19-11405512 | G→A |
| ***Pt-SPRY*-SNP03** | Chr19-11405518 | G→A |
| ***Pt-SPRY*-SNP04** | Chr19-11405534 | T→G |
| ***Pt-SPRY*-SNP05** | Chr19-11405549 | C→T |
| ***Pt-SPRY*-SNP06** | Chr19-11405550 | G→A |
| ***Pt-SPRY*-SNP07** | Chr19-11405554 | A→G |
| ***Pt-SPRY*-SNP08** | Chr19-11405570 | T→C |
| ***Pt-SPRY*-SNP09** | Chr19-11405577 | A→G |
| ***Pt-SPRY*-SNP10** | Chr19-11405583 | A→T |
| ***Pt-SPRY*-SNP11** | Chr19-11405627 | C→G |
| ***Pt-SPRY*-SNP12** | Chr19-11405628 | T→C |
| ***Pt-SPRY*-SNP13** | Chr19-11405700 | C→A |
| ***Pt-SPRY*-SNP14** | Chr19-11405937 | C→A |
